# Precision Control of Light-Responsive Nucleic Acids Modified with Photoremovable Protecting Groups for Functionalization

**DOI:** 10.1021/jacsau.5c00524

**Published:** 2025-06-19

**Authors:** Zhongqi Zhou, Hau Yi Chan, Pik Kwan Lo

**Affiliations:** † Department of Chemistry and State Key Laboratory of Marine Pollution, 53025 City University of Hong Kong, Tat Chee Avenue, Kowloon Tong, Hong Kong 999077, China; ‡ Key Laboratory of Biochip Technology, Biotech and Health Care, Shenzhen Research Institute of City University of Hong Kong, Shenzhen 518057, China

**Keywords:** ortho-nitrobenzyl, photocleavage, nucleic acids, drug delivery, gene editing

## Abstract

*o*-nitrobenzyl (ONB) groups based photoremovable protecting groups (PPGs) stand out as significant photocleavage components in oligonucleotides, owing to their straightforward synthesis, easy incorporation, and compatibility with a broad array of functional groups. These ONB-modified nucleic acids have found widespread applications in the area of biomedicine. In this perspective, we delve into the progress and study of PPGs derived from ONB for accurately controlling nucleic acid functions using light. Various chemical approaches are explored for incorporating ONB-based PPGs in different parts of single-stranded nucleic acids, including nucleobases, backbones, riboses, and phosphate groups, along with their distinct impacts on nucleic acid hybridization. Additionally, we evaluate the benefits and constraints of these alterations. Promising strategies, such as the molecular engineering of existing ONB derivatives, the integration of established PPGs with appropriate two-photon light-absorbing antennas in a modular setup, and the coupling of ONB protective groups with upconversion nanomaterials to extend their cleavage wavelengths into longer spectra are outlined and discussed. Subsequently, we systematically explore the biological applications of these light-responsive nucleic acids as versatile tools, specifically for light-triggered gene editing and as foundational elements for constructing 3D DNA-based nanomaterials for drug delivery. By leveraging the versatile chemistry of ONB alongside diverse nucleic acid modification techniques, we aim to provide an up-to-date overview while outlining existing challenges and proposing solutions within this evolving field. This Perspective seeks to foster innovation in the design of light-responsive nucleic acids within DNA nanotechnology for biomedical applications, underscoring the ongoing pursuit of novelty in this field.

## Graphical abstract

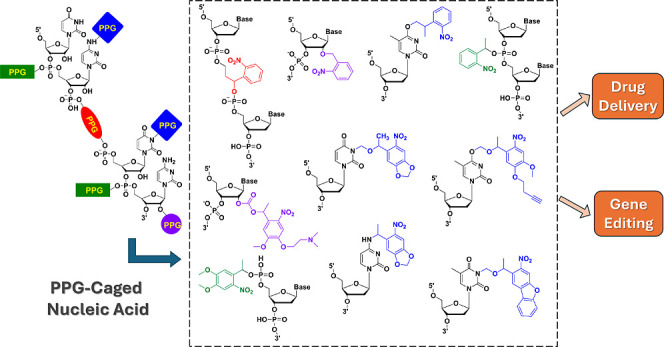

## 1 Introduction

Nucleic acids, with their programmability and predictability rooted in sequence-specific hybridization, have emerged as exceptional functional materials for constructing nanoscale molecular tools with diverse capabilities, encompassing recognition, replication, transport, and selective catalytic functions.^1^ This versatility renders nucleic acids highly appealing to researchers exploring their applications across various domains. Leveraging its molecular recognition properties, controllable nucleobase sequences, straightforward synthesis, chemical modification feasibility, and responsiveness to external stimuli, DNA has ascended as a potent scaffold for engineering intricate two-dimensional (2D) and three-dimensional (3D) soft materials.^2,3^ This adaptability allows for precise manipulation of composition, structure, and functionality.^4–6^ The base sequences of oligonucleotides, such as cytosine-rich DNA, guanosine-rich DNA, and aptamers, encode valuable functional and structural data, facilitating the rational design of stimuli-responsive DNA nanomaterials.^7,8^ Among various stimuli, light stands out as a compelling stimulus due to its cleanliness, noninvasive nature, instant manipulability, and high spatial–temporal precision, offering unparalleled control over systems.^9^ Parameters like wavelength, light intensity, and exposure duration can be adjusted to finely regulate photoresponsive activities, minimizing waste accumulation and enhancing performance efficiency. Meanwhile, utilizing light to modulate and observe processes within living systems at exceptional spatial and temporal resolutions is unparalleled. Introduction of photosensitive groups can transiently inhibit the biological activity of entities like small molecules, proteins, and nucleotides, rendering them inactive.^10^ Upon exposure to specific light wavelengths, these protective groups undergo photochemical reactions, reinstating the biological functionality of the underlying entity (Figure 1).

**Figure 1:**
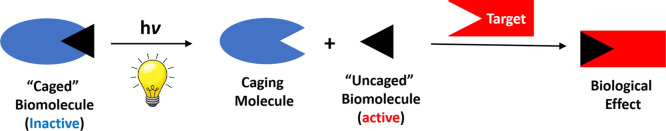
A simplified depiction of the photoregulation concept for functional biomolecule, demonstrating light irradiation of a caged biomolecule to eliminate the photolabile protecting group and rejuvenate the biological functionality of the biomolecule.

Notably, the impact of photosensitive compounds on oligonucleotides, such as aptamers, siRNAs, miRNAs, molecular beacons, and antisense oligonucleotides, is of significant importance.^11–13^ The photoregulation strategy, characterized by simplicity, cost-effectiveness, robust selectivity, high controllability, and broad adaptability, holds substantial promise for modulating protein function and gene expression.^14–16^ Various chemical approaches have emerged to incorporate photosensitive molecules into the structure of nucleic acid, as they are primarily unresponsive to light with the exception of UV–B and UV–C wavelengths. Three categories of photosensitive molecules—photocleavage molecules (including photocleavable linker and photocaged group), photoisomerization molecules,^17^ and photo-cross-linked molecules^18–20^ —are strategically placed within nucleic acid sequence to enable diverse light-driven functionalities. For example, modifying the sugar,^21,22^ phosphate^23^ or nucleobases^24^ of oligonucleotide with photocaged groups prevents hybridization with their complementary strands until the photocaged groups are removed by exposure to light. Additionally, it is possible to conjugate a photocleavable linker or photoisomerizable groups to the DNA oligonucleotide’s backbone during solid-phase synthesis, enabling site-specific strand breaking or structural/conformation switching upon light exposure.^25,26^

A pioneering study by Ordoukhanian and Taylor in the field of photoresponsive DNA materials introduced *o*-nitrobenzyl (ONB) esters into duplex DNA to enable phototriggered strand breaks.^3^ Subsequently, various irreversible photocleavable groups, including ONB derivatives, *p*-hydroxyphenacyl, TEEP–OH, aryl sulfide, nitroindole, benzophene/acetophenone, and coumarin, have been widely employed in the development of light-responsive nucleic acid tools.^27^ Among them, ONB groups stand out as prevalent photocleavage groups due to their facile synthesis, easy installation, and applicability to a wide range of functional groups. The tunable photochemical properties of ONB groups, achieved through electron-donating group modifications, enable efficient decaging with nonphotodamaging light within specific wavelength ranges, i.e. 365–420 nm. These groups have found applications in diverse systems, including aptamers,^28,29^ DNAzyme,^30–34^ negatively charged peptide nucleic acids,^35^ RNA interference (siRNA) precursors,^36^ nucleic acid for gene editing and regulation,^37–40^ splice-switching oligonucleotides,^41^ miRNA imaging and detection,^42–44^ PCR product generation control,^45^ hydrogel formation,^46–48^ DNA logic gates,^49,50^ structural switching in nanomachines,^51^ surface patterning^52,53^ and drug delivery of self-assembled DNA nanostructures.^16^ Originally designed for UV-A or visible light excitation via one-photon (OP) absorption with high energy, these compounds exhibit lower efficiency in photorelease under low-energy excitation.^54–56^ Consequently, their limited tissue penetration,^57,58^ potential harm to biomolecules like DNA^59–61^ underscore the need for engineering them to absorb light at longer (red-shifted) wavelengths.^54^ Recently, efforts are underway to extend the cleavage wavelength of ONB protecting groups to longer wavelengths, with a focus on molecular engineering, utilizing TP excitation (Figure 2),^62,63^ and conjugating to upconversion nanomaterials.

**Figure 2:**
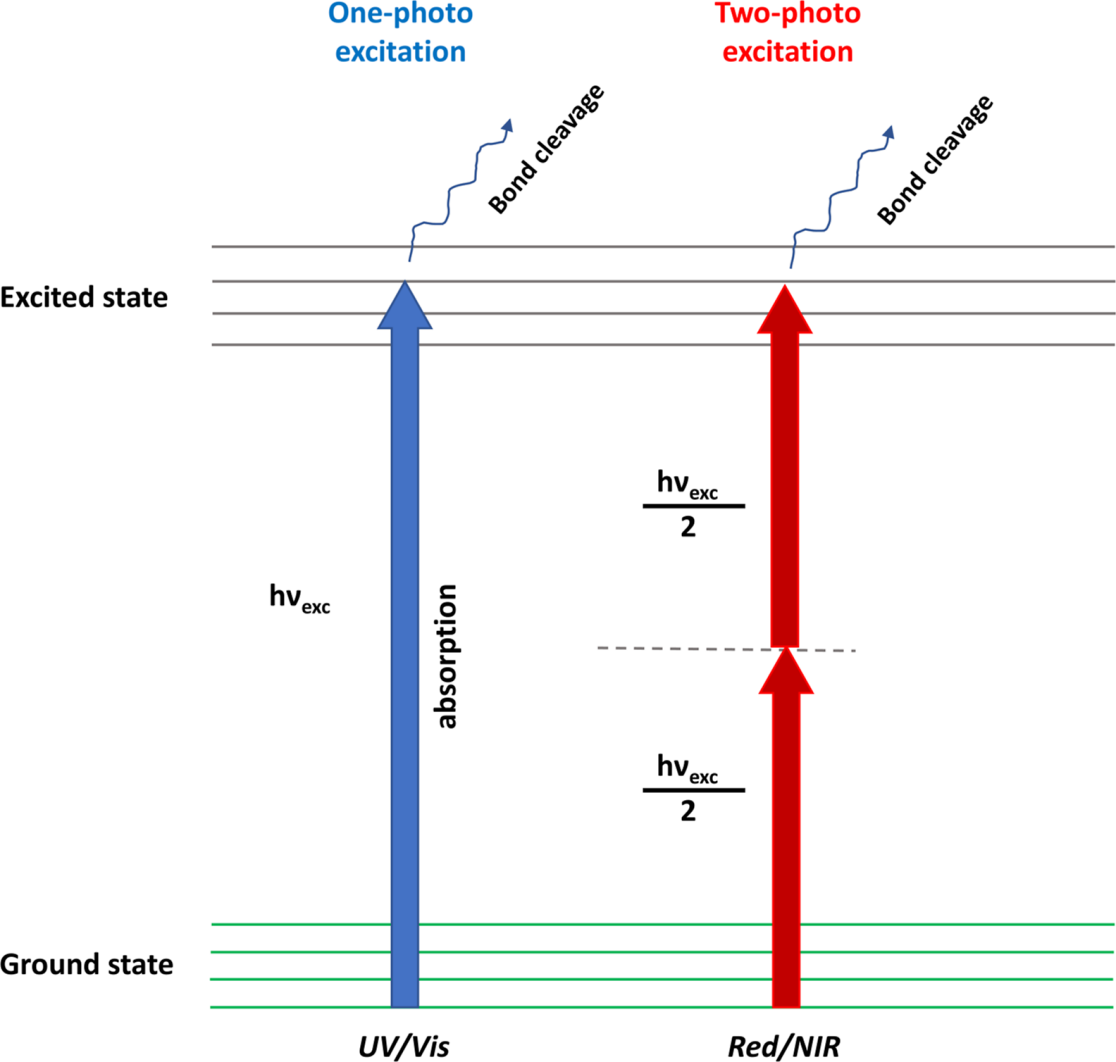
Simplified Jablonski diagram illustrating one-photon-induced and two-photon-induced photolysis processes.

By transitioning from standard OP excitation to TP excitation in the near-infrared (NIR) range (typically within the biological spectral window of 700–1000 nm), these limitations can be overcome, offering unique spatiotemporal control of photorelease.^64,65^ This perspective delves into the molecular design of ONB-based PPGs and different chemical modification strategies of nucleic acids associated with the application of this emerging photocleavage system in precise spatiotemporal control of drug delivery and gene editing. Additionally, we also focus on diverse approaches aimed at enabling longer-wavelength activation for prospective in vitro and in vivo uses. Finally, we provide an overview of the latest progressions in photosensitive DNA research. We identify prevalent gaps and challenges encountered in biological applications, offering insights to tackle these issues within the field of photoresponsive DNA nanotechnology research.

## 2 Family OF ONB PPGs for Nucleic Acid Modification

### 2.1 Classification of *o*-Nitrobenzyl PPGs

Among the most frequently utilized PPGs are derivatives of ONB groups. These compounds are favored for their straightforward chemical structure and ease of synthesis, enabling the incorporation of a diverse array of functional groups into the ONB scaffold. An overview of the commonly employed ONB derivative caged compounds is provided in Figure 3 and Table 1. They include 2-(*ortho*-nitrophenyl)-propyl (NPP) and 1-(*ortho*-nitrophenyl)-ethyl (NPE), 6-nitropiperonyl methyl group (NPM), 1-(2-nitrophenyl)­ethyl (NEP), 6-nitropiperonyloxymethylene (NPOM), propargyl-6-nitroveratryloxymethyl (PNVOM), nitrodibenzofuran (NDBF), 1-(4-(2-(dimethylamino)­ethoxy)-5-methoxy-2-nitrophenyl)­ethyl carbonyl (DMNEC), and 4,5-dimethoxy-2-nitrophenylethyl (DMNPE).

**Figure 3:**
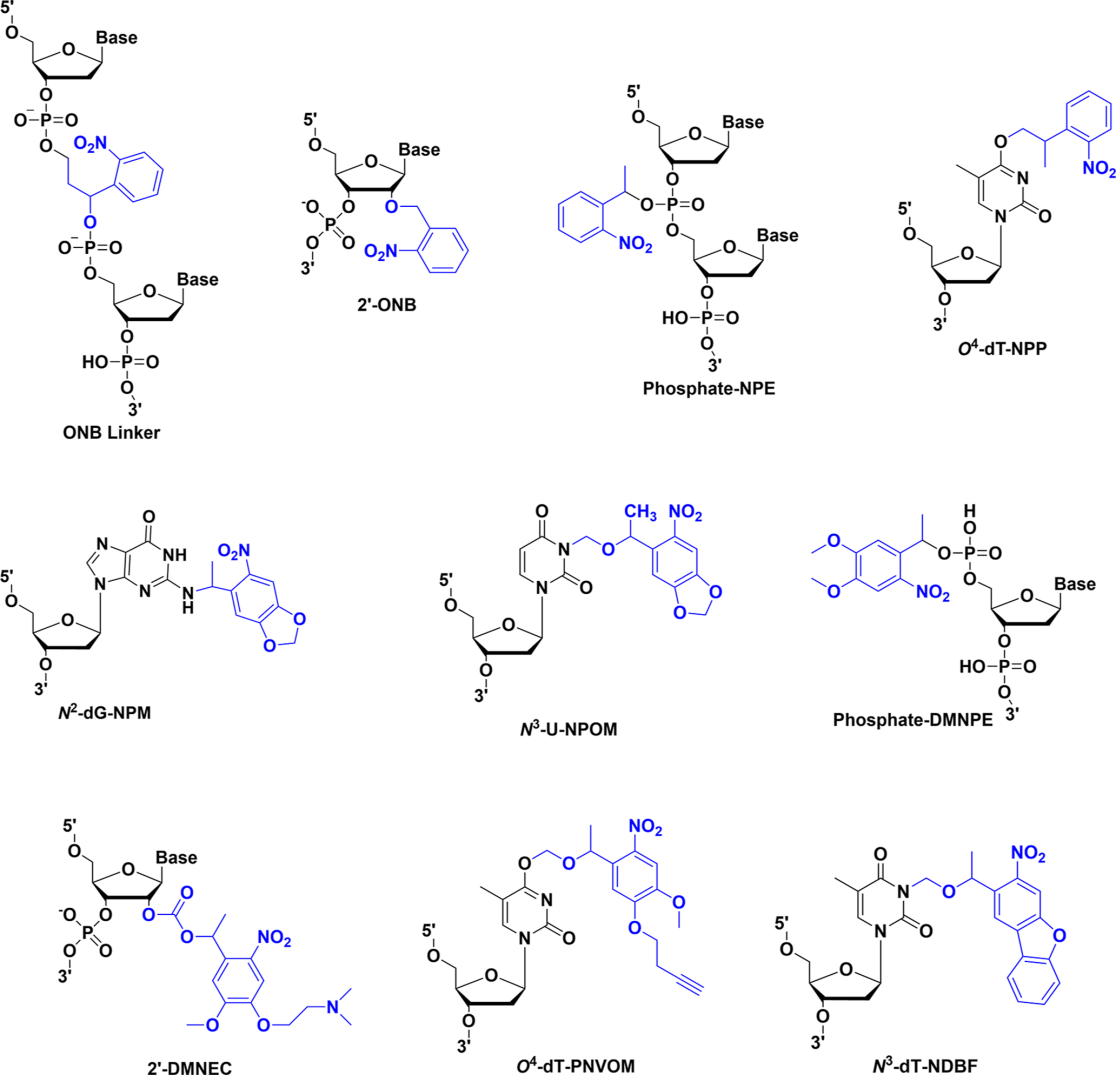
Representative structures of ONB and ONB derivatives modified on nucleic acid.

**Table 1:** Summary of Different Types of PPG-Conjugated Oligonucleotides

PPGs	position(s)	protecting/conjugating chemistry	type of oligonucleotide	excitation wavelength (nm)	light power (W)/irradiance (mW/cm^2^)	duration of irradiation (s)	references
ONB	backbone	phosphoramidite	DNA	365	35 mW/cm^2^	1800	^134^
	backbone	phosphoramidite	DNA	350 (Xe lamp)	9 mW/cm^2^	600	^135,136^
	backbone	phosphoramidite	DNA	302	6 W	720	^135,136^
	backbone	phosphoramidite	DNA	365	1200 mW/cm^2^	5	^137^
	backbone	phosphoramidite	DNA	365 (UV lamp)	35 W	30	^138,139^
	backbone	phosphoramidite	DNA	350 (xenon lamp)	300 W	600	^140^
	backbone	intramolecular cross-linking of sgRNA by RNA-CLAMP	RNA	390 (LED)	52 W	30	^141^
	backbone	phosphoramidite	RNA	345 (UV lamp)	600 W	60	^96^
	backbone	phosphoramidite	RNA	365 (LED)	6 W	30	^79^
	terminal	click reaction	DNA	365	100 mW/cm^2^	60	^142^
	terminal	copper catalyzed azide alkyne cycloaddition	DNA	302	6 W	60	^143^
	nucleobase	substitution at O4-dT	DNA	365 (LED)	100 W	1800	^86^
	phosphate	phosphoramidite	RNA	365	7 mW	240	^144^
NPE	nucleobase	substitution at O4-dT	DNA	366 (Hg lamp)	1.2 mW/cm^2^	1800	^78,83^
	nucleobase	substitution at O4–U, N4–C, N6-A, O5-G	RNA	300–400 (xenon lamp)	300 W	600	^84^
	Nucleobase	Substitution at N2-dC, O5-dG	DNA	365	100 mW	20–900	^145,146^
NPP	nucleobase	substitution at N2-dC, O5-dG	DNA	365	100 mW	20–900	^145,146^
	nucleobase	substitution at O4-dT	DNA	365 (LED)	100 W	1800	^86^
NPM	nucleobase	substitution at N2-G	RNA	365 (LED)	142 mW/cm^2^	30	^87^
	nucleobase	substitution at N2-G	RNA	365 (LED)	400 mW/cm^2^	10	^88^
NPOM	nucleobase	substitution at O4–U	RNA	365 LED	6.3 mW/cm^2^	120	^41^
	nucleobase	substitution at N3-dT	DNA	365	18.2 W	3	^81^
	nucleobase	substitution at N3-dT and N6-dA	DNA	365	NA	600–1800	^92,93^
	nucleobase	substitution at N3-dT	DNA	405 (LED)	4–8 mW/cm^2^	220	^73^
	nucleobase	substitution at N3-dT	RNA	365	3.33 mW/cm^2^	300	^147^
	nucleobase	substitution at N3–U and N1-G	RNA	365	80 mW/cm^2^	300	^148^
	nucleobase	substitution at N3-dT	RNA	365	35 mW/cm^2^	30	^149^
DMNB	terminal	thiol-halogen exchange, CuAAC click reaction, and amide coupling	MO	365	27.7 mW/cm^2^	60	^102^
	backbone	phosphoramidite	MO	365(Hg lamp)	66 mW/cm^2^	600	^99,100^
	backbone	phosphoramidite	MO	360	13 mW/cm^2^	60	^97,98^
	backbone	phosphoramidite	MO	365 (Hg lamp)	41 mW/cm^2^	10	^101^
DMNPE	phosphate	condensation reaction	RNA	365	2.16 mW/cm^2^	600	^103^
DMNEC	2′hydroxyl	acylation	RNA	365	8 W	3600	^21^
	2′hydroxyl	acylation	RNA	365	7 mW/cm^2^	300	^22^
PNVOM	nucleobase	nucleophilic substitution		365	25 W	120	^91^
NPBOM	2′hydroxyl	acylation	TNA	365	20 mW/cm^2^	60–300	^150^
NDBF	nucleobase	substitution at N6-dA and N2-dC	DNA	440	30 mW/cm^2^	1800	^78^
s-ANBP	backbone	phosphoramidite	DNA	415	200 mW/cm^2^	120	^151^
*t*-ANBP	backbone	phosphoramidite	DNA	415	200 mW/cm^2^	120	^151^
4-NB	backbone	phosphoramidite	DNA	365	200 mW/cm^2^	300	^117^
BNSMB	backbone	phosphoramidite	DNA	415 nm; 430 nm (LED)	200 mW/cm^2^	30	^125^
BNSF	backbone	phosphoramidite	DNA	415 nm; 430 nm (LED)	200 mW/cm^2^	30	^125^

Among these ONB and it derivatives modified on nucleic acid, the Deiters group notably contributed to the development of NPOM-caged DNAs for a broad array of in vitro and in vivo biological applications. They successfully synthesized NPOM-caged 5′-dimethyoxytrityly (DMT)-protected thymidine/guanosine/uridine phosphoramidite for subsequent incorporation, into synthetic DNAs/RNAs. These NPOM-modified nucleotides can be efficiently decaged upon exposure to 365 nm light thereby regulating DNA and RNA base-pairing abilities for applications such as controlling RNA interference,^66^ DNAzyme activity,^67,68^ gene promoter activity,^38^ polymerase chain reaction.^69,70^ and restriction endonuclease activity,^71^ as well as influencing TLR9 in immune responses,^72^ and modulating *sox31*mRNA splicing through splice switching in zebrafish embryos.^41^ More recently, Tavakoli et al. also demonstrated that NPOM-modified DNA can be recognized by the Rad4/XPC protein as a specific substrate. This specific binding was found to be reversible through light-induced cleavage of the NPOM group from DNA in a dose-dependent manner.^73^ It is pertinent to note that the release kinetics of ONB and its derivatives tend to be gradual, and postirradiation, the generation of nitrosoaldehyde byproducts may engage in unfavorable interactions with amines. Such interactions have the potential to adversely affect neighboring proteins, thereby possibly eliciting cytotoxic effects.^74^

### 2.2 Photophysical and Thermostability Properties

The stability and absorption characteristics of ONB-based caged molecules can be effectively tuned by modifying substituents on the benzylic ring. Specifically, introducing an electron-donating group at the meta position or an electron-withdrawing group at the para position relative to the nitro group induces a red-shift in the absorption spectrum, enabling photocleavage with longer-wavelength light. Rodrigues-Correia et al. conducted a DNA melting study showing that oNB derivatives like NPP, NPE, NPOM, and NDBF generally reduce the melting temperature (*T*_m_) of 15-mer DNA duplexes by 6–16 °C, with variations depending on the sequence.^75^ The thermal stability was also influenced by the stereochemistry of the NPE group: the (*S*)-NPE isomer lowered the *T*_m_ by 9.2 °C, while the (*R*)-NPE resulted in a smaller 4.8 °C decrease. Although both enantiomers preserved Watson–Crick base pairing, differences in their interactions with neighboring bases led to distinct effects on duplex stability.^76^ Tavakoli et al. found that incorporating NPOM into a 24-mer DNA duplex decreased the *T*_m_ by approximately 7 °C. Following photocleavage, the Tm returned to the level of the unmodified duplex.^73^ Molecular dynamics simulations suggested that NPOM adopts a syn-conformation, positioning itself in the DNA’s major groove. When three NPOM groups were introduced across a 14-base-pair region, the *T*_m_ dropped by about 30 °C.^77^ In RNA duplexes, the impact of NPOM modifications on thermal stability depended on both the number and position of modifications.^66^ Additionally, NDBF modifications at N^4^-dC and N^6^-dA in 15-mer DNA duplexes reduced *T*_m_ by 16 and 12 °C, respectively. This is greater decreases than those from NPE at the same positions (8 and 6.2 °C).^78^ Additionally, photocleavage duration varies based on light intensity, ranging from seconds to minutes with power levels from milliwatts to watts.^71,73,79,80^ In one study by Stephanopoulos et al., ∼85% of NPOM-caged DNA was cleaved within 3 s using an 18.2 W UV lamp.^81^

## 3 Locations of PPGs in Nucleic Acids and Their Roles

The positions where photoactivatable or photolabile groups can be situated are outlined in Figure 4A. They can be linked and integrated into various locations such as nucleobases (N), backbone (B), ribose (R), and phosphate groups (P) within a single-stranded nucleic acid.

**Figure 4:**
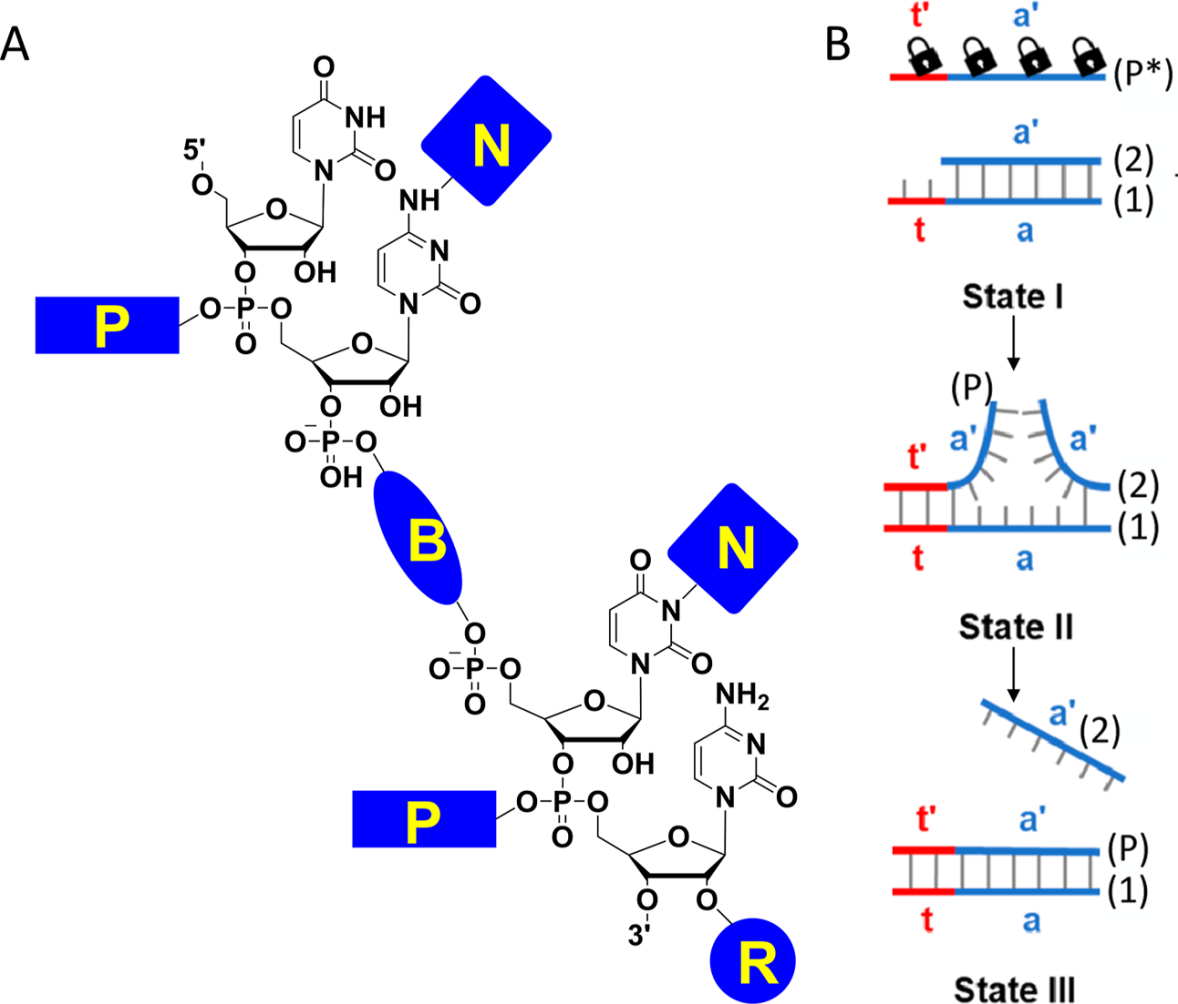
(A) Schematic diagram illustrating the four positions of PPGs in nucleic acids: (N) attached to nucleobases, (B) inserted in the backbone, (R) attached to the 2′-hydroxyl of ribose, (P) attached to the phosphate group. (B) Schematic diagram demonstrating the use of nucleobase-caged oligonucleotides (P*) for light-induced toehold-mediated dissociation of a duplex nucleic acid. Reproduced from ref 11 Copyright 2023 American Chemical Society.

### 3.1 Attachment to Nucleobases

By obstructing crucial hydrogen bonding sites within the nucleobases of nucleic acids, the formation of Watson–Crick duplexes with complementary partners is hindered, thereby impeding the reactivity of single-stranded nucleic acids toward strand hybridization. Upon exposure to light and subsequent cleavage of the photoactivatable group from the shielded nucleobases, the hydrogen bonding functionality is reinstated, enabling the activation of the strand for duplex formation. Early examples include nucleotides modified with NPP^75,82,83^ and NPE^78,83,84^ caged nucleotides—among the first ONB-based caged nucleobases.^85^ These caged modifications have been used to regulate biological interactions; for instance, ONB-dT and NPE-dT were employed to prevent the MutS mismatch repair protein from binding to a DNA bulge. Upon photoirradiation, the caging group was removed, restoring MutS binding and subsequently enabling transcriptional inhibition by T7 RNA polymerase.^86^

Subsequently, NPM was introduced onto G,^87,88^ while its 6-nitropiperonyloxymethylene (NPOM) analogs on N^3^-dT, N^3^–U and N^1^-dG. This modification aimed to enable longer decaging wavelengths compared to those used for ONB and to enhance stability in aqueous solutions across various pH levels.^14^ These newly introduced groups exhibit a decaging wavelength falling within the UV-A range, typically at 360–366 nm, making them orthogonal to commonly utilized fluorescent proteins. This characteristic allows for direct interfacing with numerous reporter systems,^10^ with minimal cellular and organismal toxicity upon light exposure.^89,90^ Further variations of ONB-based photocleavable motifs, such as PNVOM^91^ and NDBF group,^78^ have also been documented in recent studies.

Liu et al. engineered a self-assembled DNA nanotweezer for light-induced irreversible switching.^81^ The control sequence responsible for regulating the switching of self-assembled DNA nanotweezers is initially embedded within the DNA nanostructure. However, photocage NPOM molecules are attached to the nucleobases of the control strand before activation, impeding their ability to hybridize with the complementary strand. Upon exposure of the nanotweezers to 365 nm light, the photocage molecules detach from the nucleobases of the control strand, restoring their hybridization capability and initiating the transition of the nanotweezers from a closed state to an open state. Compared to external stimulus-induced triggering, this method of switching using self-assembled DNA nanostructures offers the advantages of quicker response times and enhanced temporal and spatial precision.

Evidently, photocaged oligonucleotide bases can be effectively utilized for light-induced toehold-mediated strand displacement within duplex nucleic acid systems. As shown in Figure 4B, a partially duplex nucleic acid containing a short single-stranded toehold (T) is combined with the photocaged invading strand (P*), engineered to possess sequences that complement this toehold. Initially, at state I, the toehold-mediated displacement of strand (2) by strand (P*) is hindered due to the presence of photoactivatable groups on strategic nucleobases in strand (P*). Upon exposure to light, these groups are cleaved, yielding the uncaged strand (P), which proceeds to displace strand (2) upon recognizing the toehold region, forming the thermodynamically stable (P)/(1) duplex. Prokup et al. adapted a similar concept to devise a light-triggered AND gate employing a gate complex comprising a fluorophore strand (*G*_F_), a quencher strand (*G*_Q_), a DNA strand with a toehold (*G*_T_), a trigger strand modified with NPOM caged groups on thymidine nucleotides (A_4_), and an uncaged DNA strand (B_0_) (Figure 5A).^92^ To activate the gate, simultaneous irradiation with 365 and 532 nm light is applied, leading to the deprotection of A_4_ and facilitating the toehold displacement cascades alongside B_0_. This process culminates in the removal of *G*_Q_ from *G*_F_, resulting in the restoration of the fluorescent signal as the output. Subsequently, the same group applied hybridization chain reaction to amplify the signals for DNA computation.^93^

**Figure 5:**
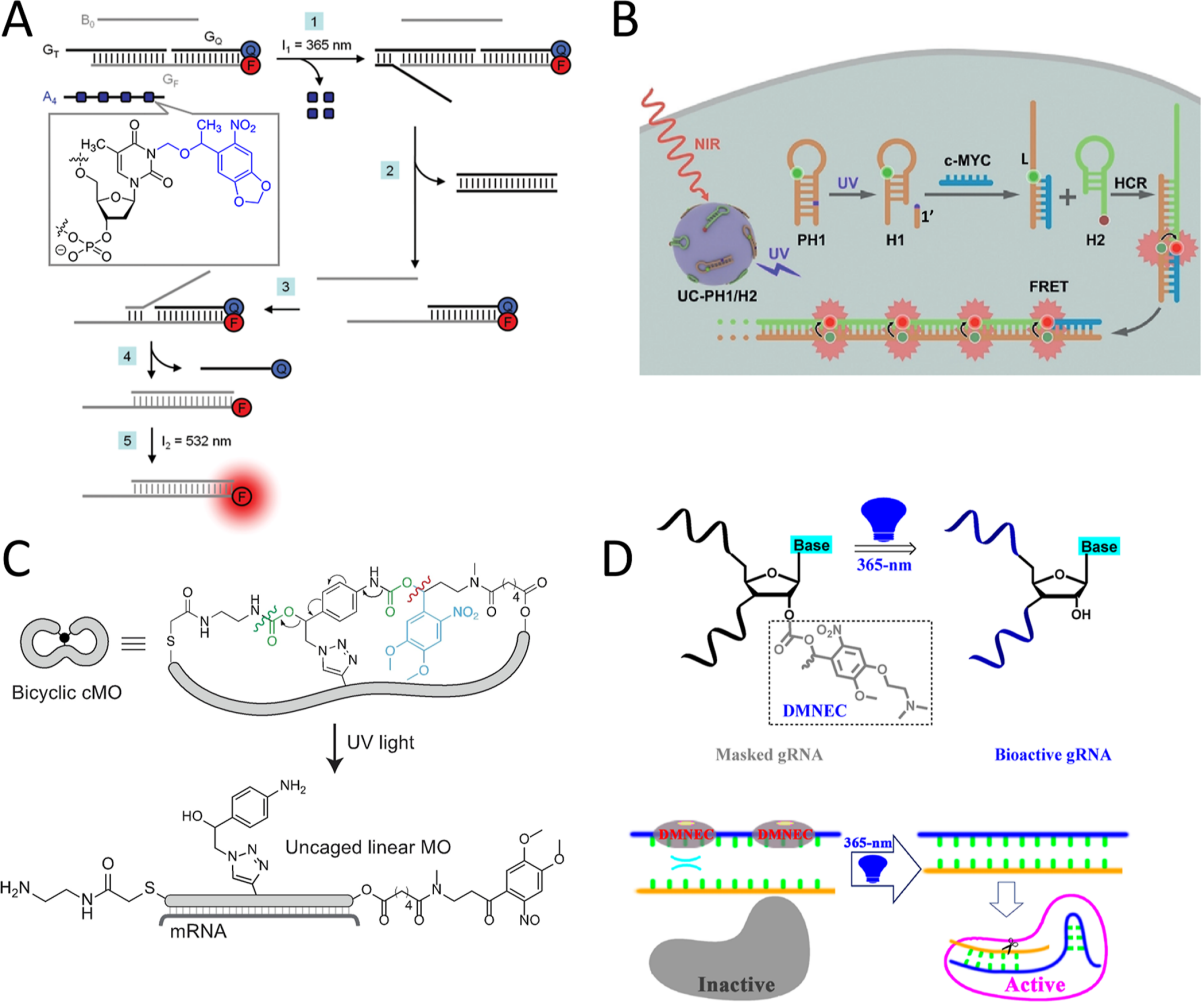
(A) Uncaging the trigger strand modified with NPOM caged groups on thymidine nucleotides (A4) to facilitate the toehold displacement cascades for operating a DNA-based AND gate. Reproduced from ref 92. Copyright 2012 American Chemical Society. (B) Photoactivated toehold-mediated strand displacement using ONB-modified hairpin structures in conjunction with HCR technique for spatiotemporally resolved imaging of c-MYC mRNA with signal amplification. Reproduced with permission from ref 94 Copyright 2019 John Wiley and Sons. (C) The uncaging mechanism of bicyclic cMO and its subsequent hybridization to the targeted mRNA after light irradiation. Reproduced with permission from ref 102 Copyright 2022 John Wiley and Sons. (D) Application of the postsynthetic polyacylation reaction to conceal gRNA via its 2’–OH group for CRISPR function inhibition. The CRISPR system was reactivated by phototriggered release of the DMNEC-based PPGs. Reproduced from ref 22 Copyright 2020 American Chemical Society.

### 3.2 Backbone Insertion

Nevertheless, the presence of short single-stranded toeholds in complex biological systems may impede their efficiency, as they remain readily available for hybridization with invading strands. Consequently, safeguarding the toeholds within duplex structures, followed by unveiling them upon irradiation, is strongly recommended. For instance, Chu et al. employed the concept of photoactivated toehold-mediated strand displacement using ONB-modified hairpin structures in conjunction with a hybridization chain reaction (HCR) to detect and visualize target c-MYC mRNA in MCF-7 cells (Figure 5B).^94^ The hairpin structure (PH1) is designed to incorporate a photocleavable ONB linker within one of its stem sections. Upon cleavage of ONB, the hairpin strand divides into a longer strand, L, and a shorter strand, 1′, which is too brief to form a stable duplex with L. Consequently, 1′ dissociates from L, revealing a single-stranded toehold in the L strand. This region is tailored to hybridize with the complementary domain on the target mRNA, initiating strand displacement and the unfolding of the hairpin structure, driven by the eventual creation of more stable H1:c-MYC products. The introduction of H2 facilitated signal amplification in this study. The toehold can also be concealed within the loop region of a self-complementary DNA hairpin, akin to the fluorescent probe design reported by Huang et al.^42^ Following uptake by cells in its intact state, the stability of the hairpin duplex (CPF) stem hinders access to the masked toehold. Upon a phototriggered strand breakage event, the hairpin loop opens, making the toehold accessible for hybridization with a target MnSOD mRNA in living cells. ONB, NPP, and NPE groups have been widely utilized as photocleavable linkers within the oligonucleotide backbone or protecting group of phosphate to control gene expression^95^ and gene editing,^79,96^ and RNA-cleaving activity.^33^

Besides DNAs, backbone insertion of photocleavable molecules on morpholino oligonucleotides (MOs) represent synthetic tools that lay dormant until activated by specific wavelengths of light, enabling light-triggered gene silencing within living organisms. The initial development of ONB-based photocaged morpholinos (cMOs) featured a hairpin configuration, where a shorter inhibitory MO prevented the longer antisense MO from binding to its target mRNA.^97,98^ Subsequent advancements introduced various MO-based caging techniques, such as employing duplex structures between the entire MO and cleavable complementary oligonucleotides for gene expression control in zebrafish.^99,100^ Moreover, cyclic cMOs were innovatively designed by linking the oligonucleotide termini with a photocleavable linker, introducing curvature to impede cMO-RNA hybridization due to limited conformational flexibility within oligonucleotide duplexes.^101^ However, these pioneering methodologies may face practical constraints. Crafting hairpin cMOs demands precise adjustment of inhibitor binding energetics to minimize basal activity, with potential toxicity concerns arising from the inhibitory MO itself. Similarly, the complementary oligonucleotides released by photoactivated duplex cMOs could pose toxicity risks. In contrast, during cMO cyclization, unintended intermolecular connections may form, necessitating molecule purification to avert adverse impacts on experimental outcomes, thus adding complexity to experimental procedures. Additionally, cyclic cMOs exhibit elevated dark-state activity. To tackle these challenges, Pattanayak et al. recently introduced a groundbreaking bicyclic MO oligonucleotide structure utilizing a trifunctional cross-linker (Figure 5C).^102^ They engineered a new self-immolative incorporating a single 4,5-dimethoxy-2-nitrobenzyl (DMNB) group. In this innovative design, the DMNB component was strategically linked to a masked hydroxymethylaniline scaffold via a carbamate linkage, with the methylenetriazole moiety in the benzylic position intended to enhance the reaction rate. Additionally, three distinct reactive groups—a carboxylic acid-functionalized DMNB group, an azide-bearing hydroxymethylaniline, and a chloroacetamide—were carefully chosen to undergo successive reactions with a linear MO containing an amine, internal alkyne, and thiol, respectively. Upon exposure to light, the DMNB group underwent cleavage (highlighted in red), prompting the caged MO oligonucleotide to initiate a spontaneous 1,6-elimination cascade reaction (shown by arrows), leading to the linearization of the caged oligonucleotide and the release of CO_2_ (indicated in green).

### 3.3 Attachment to 2′-Hydroxyl

Given that the 2′-OH group on the ribose of RNA displays notable nucleophilicity owing to its low p*K*_a_, it is advisible to modify it with PPGs to prevent its structure from cleavage at specific sites and inhibit premature folding and interactions with other molecules before its intended biological activity. The DMNEC moiety has been employed for acylating the 2′-hydroxyl of RNA. Velema et al. introduced a photocloaking approach to shield the 2’–OH groups within lengthy, biologically significant RNA segments, preserving their structure and impeding their functions.^21^ They devised photocloaking agents comprising an active acyl group, an *o*-nitroveratyl moiety, a water-soluble dimethylaminoethyl group, and a 2-chloroimidazole leaving group for the polyacylation reaction. Upon exposure to 365 nm light, these blocking groups are removed, reinstating the activity of a hammerhead ribozymeMore recently, Wang et al. applied a similar concept by conjugating a DMNEC derivative to shield the guide RNA (gRNA) and suppress CRISPR functions (Figure 5D). Upon UV-A light irradiation, this process triggers the removal of the masked gRNA, thereby restoring CRISPR function for gene editing in human cells.^22^

### 3.4 Attachment to Phosphate Group

The covalent bonding of a photolabile group to a nucleic acid duplex like RNA can physically obstruct the initial interaction between siRNA and RISC, thereby halting the degradation ofits target mRNA. Only upon exposure to light would the native siRNA be liberated. Kala et al. altered the siRNA with a DMNPE group by reacting the precursor DMNPE hydrazone with MnO_2_ to produce diazo-DMNPE, which subsequently reacts with the phosphate groups in siRNA.^103^ Tang’s group introduced a novel technique to modify the phosphate groups in siRNA by synthesizing a complete set of four NEP shielded nucleoside phosphoramidites (dA^0^, dG^0^, dC^0^, and dT^0^) from 2-cyanoethyl-1-(2-nitrophenyl)­ethyl-*N*,*N*′-diisopropyl-phosphoamidites (N^0^).^95^ This approach enables highly specific integration of photocleavable molecules into siRNA strands through solid-phase synthesis.

## 4 4.Strategies for Achieving Longer Wavelength Activation

### 4.1 Extending π-Conjugation and Introducing Electron-Withdrawing Groups (A) and Electron-Donating Groups (D) in Push–Pull Systems

Several studies have contributed to identifying the critical factors that define molecular engineering guidelines for enhancing 2PA in dyes.^104^ Specifically, “push–pull” systems (*D*–π–*A*) containing strong electron-donor groups (*D*) and strong electron-withdrawing groups (*A*), connected by polarizable π-conjugated bridges, exhibit significant two-photon absorption (TPA) responses due to the intramolecular charge transfer (ICT) phenomenon.^105^ The molecular symmetry also plays a key role in determining the TPA response.^106^ Regarding OP absorption, the efficiency of PPGs for two-photon uncaging (TPU) is often quantified by the TPU sensitivity δ_u_ (in Goeppert-Mayer (GM)), which is the product of the two-photon absorption cross-section σ_2_ and uncaging quantum yield Φ_u_.^107^ Gug et al. reported the 3-(2-propyl)-4′-methoxy-4-nitrobiphenyl (PMNB) cage as an efficient TP photoremovable protecting group for glutamate with δ_u_ of 3.1 GM at wavelength of 740 nm.^108^ Its uncaging cross-section has been increased in comparison with the well-known methoxynitrobenzyl platform by extending the π system. *p*-dialkylaminonitrobiphenyl (ANBP) derivatives, features a donor–acceptor biphenyl core and a dimethylamino substituent at the para position,^109,110^ exhibited a dramatic increase of the δ_u_ value from 0.098 to 11 GM, resulting in remarkable photophysical and photochemical properties under both single and two-photon excitation.^111,112^ for efficient release of carboxylate.^108^ phenol,^113^ and phosphate groups.^109^ Other studies also pointed out that TPA cross sections of oligomers can be enhanced by biexcitonic coupling between two weakly conjugated monomers.^114–116^ Despite the promising features of these modified PPG derivatives, their chemical functionality has posed challenges when it comes to backbone insertion along DNA strands. Presently, these derivatives are monofunctional, which limits their applicability due to experimental and synthetic complexities. Recently, our group focused on designing new π-conjugated photocleavable linker for DNA conjugation. For example, we successfully synthesized an extended π-conjugated system of ONB, namely 4-nitro-4′-phenoxy-1,1′-biphenyl (4-NB) with different functional groups including trityl-protected and phosphoramidite moieties via typical chemical reactions. These non-nucleosidic phosphoramidite-based photoactivatable molecules can then be conjugated with nucleic acids (4-NB LS) via our well-established standard cyanoethylphosphoramidite chemistry for DNA self-assembly (Figure 6A). Following self-assembly of 4-NB functionalized DNAs into 3D DNA nanotubes (TPNTs), these tubular structures would lose their rigidity and become flexible tubes with curving features upon near NIR light irradiation.^117^ (Figure 6B). Additionally, we applied photoactivated, triangular-shaped DNA nanodevices to imitate the OR gate operation.^118^ Both one-photon (OP) sensitive molecule 1-(2-nitrophenyl)-ethyl derivative (2-NP) and TP sensitive molecule 4-NB were introduced to the DNA-based nanosystem. We revealed the use of NIR and UV-A as stimuli to remotely trigger the responses of these devices (Figure 6C). This would lead to restore the detectable fluorescence signals of dyes (outputs). This nanodevice can be recycled to its original state and performed repeatedly. We envision this work provides a step forward employing light-responsive nucleic acid–based nanostructures as versatile nanomaterials in optical communication and nucleic acid–based computing.^119^ However, the 4-NB molecule is also sensitive to one-photon cleavage because of its UV/vis absorption maximum present at 360 nm. Its two-photon cleavage efficiency is also highly limited due to its small TPA cross-section value and low quantum yield of the photochemical reaction.^54^ Longer NIR irradiation time is needed but this would generate a large amount of heat in the system. Although these modified PPGs were engineered to absorb NIR light, such modifications often come at the cost of reduced quantum yield and can unpredictably affect both TP absorption cross-section and uncaging efficiency, sometimes leading to counterproductive outcomes.^120^

**Figure 6:**
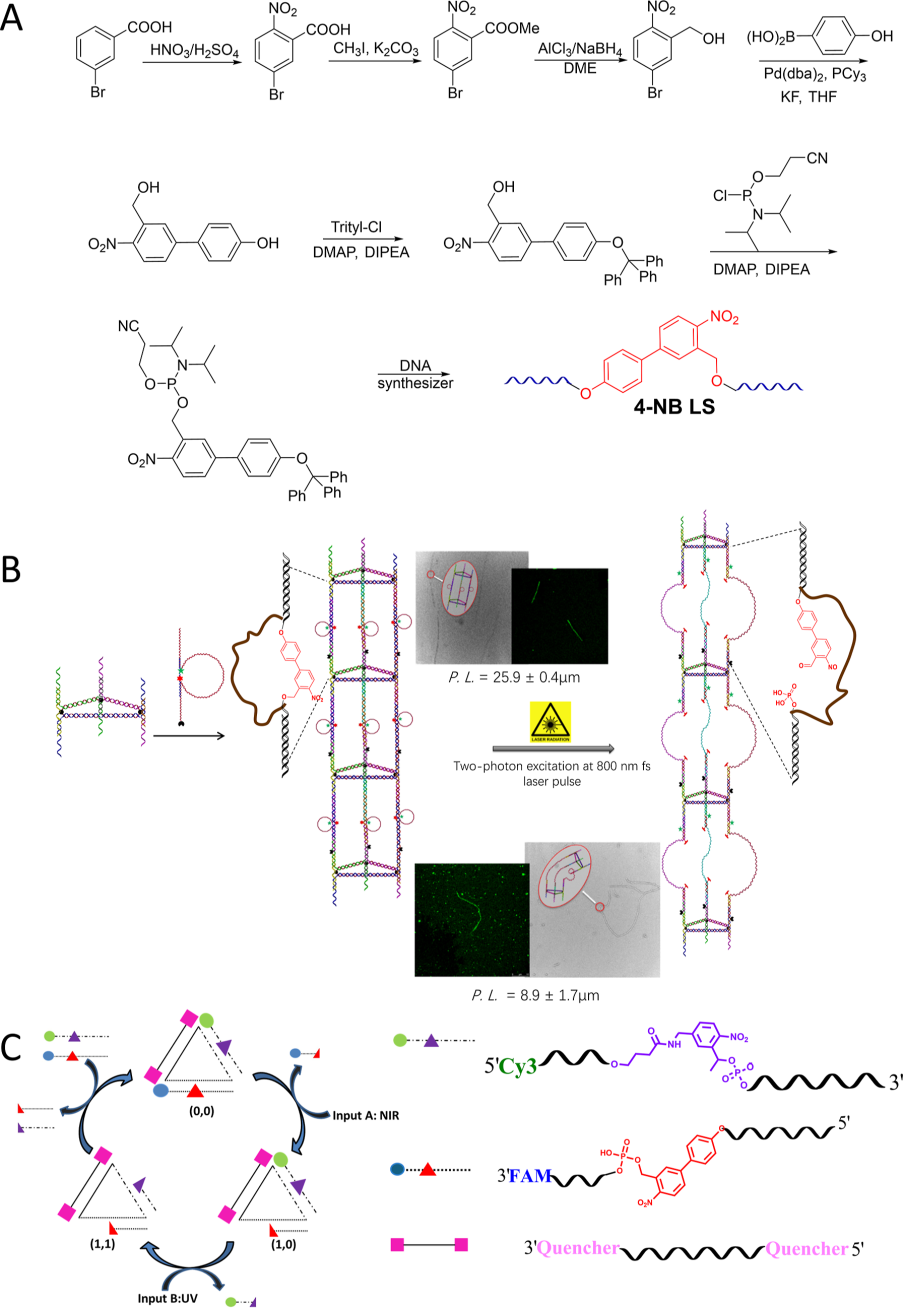
(A) Synthetic scheme depicting the trityl-protected 4-NB phosphoramidite for solid-phase synthesis of 4-NB LS and self-assembly of 4-NBHP ds-LS1 in a hairpin structure. Reproduced with permission from ref 117. Copyright 2015 John Wiley and Sons. (B) Formation of linear TPNT and its two-photon photocleavage under NIR excitation for 2 h at 86 mW laser power. The conformational alteration of TPNT was analyzed using TEM and confocal fluorescence imaging. Reproduced with permission from ref 117 Copyright 2015 John Wiley and Sons. (C) Development and functionality of a reversible DNA logic gate platform through UV-A and NIR light excitations. Reproduced with permission from ref 118 Copyright 2015 John Wiley and Sons.

### 4.2 Two-Photon Fluorophores-Based Photocages (by Tandem Strategy)

An alternative and increasingly prominent approach for developing versatile photon-sensitive caging systems involves combining known PPGs with a suitable two-photon light-harvesting antenna within a modular architecture.^106^ This strategy has become a central focus for synthetic chemists aiming to improve the efficiency of such systems. Gug et al. reported the molecular engineering of new linear caging platforms, (4,4′-bis-{8-[4-nitro-3-(2-propyl)-styryl]}-3,3′-dimethoxybiphenyl (BNSMB), which was composed of two vinylogues of PMNB linked together to take advantage of a possible interaction between the two monomers.^121^ A double bond was introduced in the system to improve its solubility in organic solvents. Indeed, this increase in the length of conjugation is beneficial to the TPA properties with δ_u_ of 0.9 GM at wavelength of 800 nm, but can be detrimental to the uncaging quantum yield, as it might induce photochemical side reactions. The methoxy group was moved from the para to the ortho position, allowing the two monomers to be linked by a single C–C bond. This structure can be considered as a biphenyl core with two electron-donating methoxy groups, surrounded by two styrenic π systems bearing electron-withdrawing nitro groups. The nitro groups also play a fundamental role in the uncaging process, as they are involved in the mechanism of the photochemical reaction. They further designed a caging platform called (2,7-bis-{4-nitro-8-[3-(2-propyl)-styryl]}-9,9-bis-[1-(3,6-dioxaheptyl)]-fluorene (BNSF) with a fluorenyl central core and substituted in the 9-position by 1-(3,6-dioxaheptyl) chains to increase aqueous solubility. This BNSF molecule based on a dipolar architecture, in which the methoxy groups were removed and the interaction between the two acceptor groups was enforced by retaining the planarity of the central core. It is important to note that BNSF exhibited δ_u_Φ_u_ of 5 GM at wavelength of 800 nm, which may be beneficial to efficient photolysis reaction.^121^ Additionally, such dipolar or quadrupolar structures is promising for the uncaging process, as they carry two photoremovable moieties that can photorelease two biomolecules per cage. Therefore, the take home message here is that a linear or planar TPA chromophore composing of two electron-donor or electron-acceptor substituents linked to a conjugated central core is highly important.^122^ In these studies, BNSF and BNSMB derivatives were reported as efficient photoreleasing tools of glutamate and polymers via ester linking.^121,123^ To further conjugate to nucleic acid, typical DMT protecting group and phosphoramidite moiety should be integrated to the BNSF and BNSMB molecules. As reported, the synthesis of BNSF or BNSMB starts from 2,7-dibromofluorene or 4,4′- diiodo-3,3′-dimethoxybiphenyl compounds, respectively. These starting materials were reacted with 3-ethyl-4-nitrostyrene in 1:2 ratio, then followed by deprotonation and nucleophilic addition on formaldehyde to generate symmetrical and highly conjugated products. We attempted to apply the same synthetic strategy to further couple the 4,4′-dimethoxytrityl group and phosphitylation units on these dihydroxyl-functionalized bis­(stilbene) molecules BNSF and BNSMB. Unfortunately, the synthetic yields of these resulting phosphoramidites were extremely low (∼5%). Indeed, the chemical syntheses reported by Nicoud and co-workers^121^ cannot be effectively adapted to the preparation of phosphoramidites for oligonucleotide conjugation. To address these limitations, we applied another new approach to synthesize two new phosphoramidites based on BNSF and BNSMB motifs for further backbone insertion along DNA strands via standard cyanoethylphosphoramidite chemistry. The detailed synthetic procedures and schemes are shown in Figure 7A. The BNSF- and BNSMB-functionalized DNA oligonucleotides showed fast and efficient one-photon uncaging properties at 420 nm by cleavage of photolabile bonds of NPPOC moiety,^124^ resulting in short pieces of DNA fragments.

**Figure 7:**
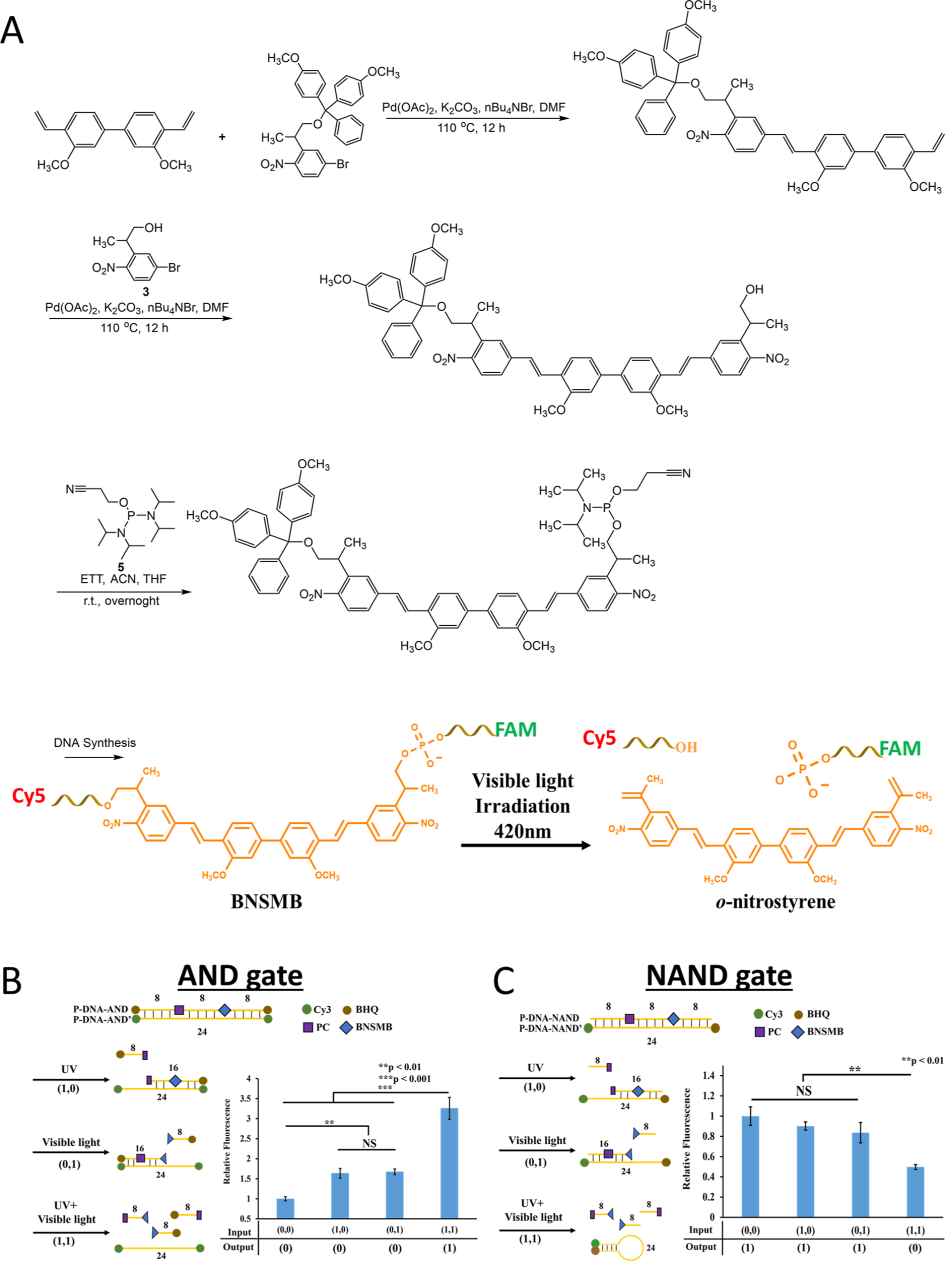
(A) Synthesis pathway of the trityl-protected BNSMB phosphoramidite for solid-phase production of BNSMB-functionalized DNA oligonucleotides and its photon-induced cleavage under 420 nm excitation. Chematic representations and relative fluorescence levels for (B) AND gate and (C) NAND gate under various light exposures. Reproduced from ref 125 Copyright 2023 American Chemical Society.

BNSF-functionalized DNAs showed two-photon uncaging activity at 800 nm but with limited uncaging efficiency. This BNSF linker, along with the standard ONB group, was used to construct photoregulated DNA devices as duplex structures. Through careful adjustments in the number and positioning of photocleavable molecules, fluorophores, and quenchers, a variety of DNA devices that functioned as Boolean logic gates (AND, OR, NOR, and NAND gates) in response to different light wavelengths were created (Figure 7B,C).^125^ However, the bifunctionalized BNSMB faced challenges due to its limited sensitivity for reliable detection at 800 nm, requiring prolonged irradiation time at 415 nm, which could lead to excessive heat generation within the system.

### 4.3 Upconversion Materials-Based Photocages

Upconversion materials possess the ability to convert low-energy photons into higher-energy ones. In the context of UV/visible light-sensitive photocage molecules, these materials facilitate the conversion of NIR light, which is both safe and capable of penetrating deep tissues, into UV-A light. This UV-A light, in turn, can initiate the cleavage of ONB molecules. Leveraging upconversion materials enables precise activation of photocages within deep tissues, addressing the limitations associated with the constrained reach and potential risks of direct UV-A exposure. In 2012, Jayakumar et al. pioneered the development of an upconversion nanoparticle (UCNP) featuring a NaYF_4_ core coated with an amorphous, mesoporous silica layer.^126^ The lanthanide-ion doped UCNP emitted light at 350 nm, aligning with the absorption profile of DMNPE. Consequently, DMNPE was utilized to cage siRNA and plasmid DNA for subsequent loading into the silica pores of the UCNPs (Figure 8A). Following successful in vitro validation of the DMNPE uncaging process in B16-F0 cells postexposure to 980 nm light, UCNPs carrying caged GFP were administered into mice, enabling effective in vivo tracking of green fluorescence protein (GFP) fluorescence. Recently, Yang et al. introduced a Zn^2+^-specific NIR DNAzyme nanoprobe for monitoring metal ions in real-time within early zebrafish embryos and larvae.^127^ They engineered UCNPs with DNAzymes modified by a nitrobenzyl group at the 2′-OH of adenosine ribonucleotide. These UCNPs convert deeply penetrating 980 nm NIR light into 365 nm UV emission, effectively removing the photolabile protecting group and activating the DNAzyme. Upon encountering Zn^2+^ ions, the activated DNAzyme cleaves its substrate, releasing a previously quenched, fluorescent FAM-labeled fragment and restoring its fluorescence signal. This DNAzyme-UCNP setup successfully facilitated Zn^2+^ detection within the NIR biological imaging range, with visible fluorescence output observable in both live cells and zebrafish embryos (Figure 8B). Li’s group also expanded upon this approach to detect ATP,^128^ mitochondrial miRNA,^129^ enzymes^130^ and pH changes^131^ in live cells using a similar UCNP system functionalized with ONB-modified Furthermore, various UCNP-based photocaging systems have been developed, such as folate caged with 2-nitrobenzylamine^132^ and d-luciferin caged with 1-(2-nitrophenyl)­ethyl.^133^ Notably, all these systems have demonstrated successful in vivo applications, underscoring the promise of NIR-to-UV UCNPs as a secure and efficient platform for surpassing the constraints of conventional UV-triggered photocages.

**Figure 8:**
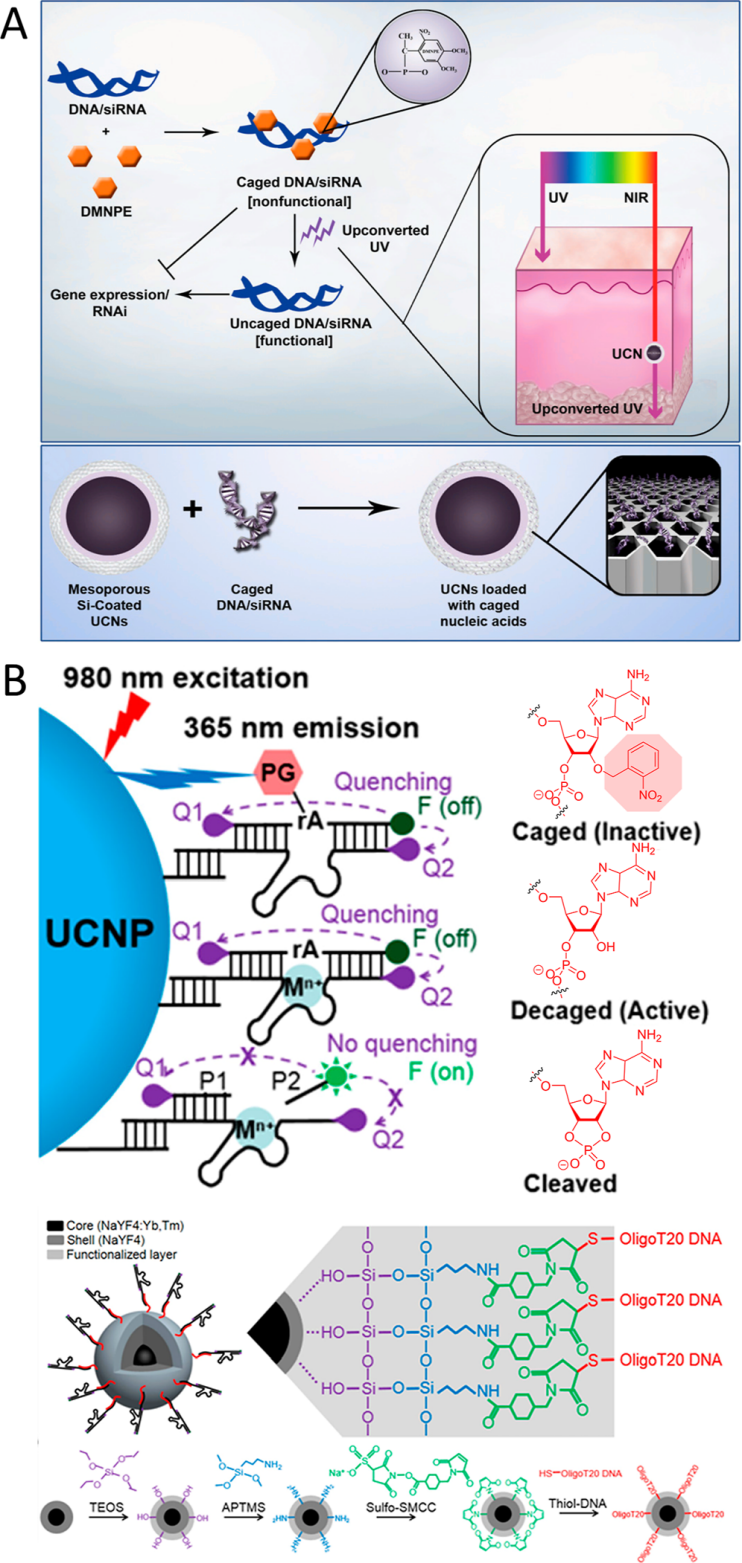
(A) Loading of DMNPE-caged plasmid DNA and siRNA onto UCN particles, subsequently uncaged using upconverted UV-A light. Inset highlights the variance in penetration depth between UV-A and NIR light in skin tissues. Reproduced with permission from ref 126 Copyright 2012 PubMed. (B) Schematic illustration demonstrating the fabrication of a photocontrollable UCNP serving as a DNAzyme-based nanosensor responsive to Zn^2+^. Reproduced from ref 127 Copyright 2018 American Chemical Society.

## 5 Biological Applications

### 5.1 Drug Delivery

In recent developments, photocleavable DNA strands have been successfully harnessed to design light-responsive microcapsules capable of releasing therapeutic cargo upon photoirradiation, enabling precise spatiotemporal control of drug release (Figure 9A).^134^ These microcapsules were crafted through the sequential deposition of photocleavable DNA layers onto a precoated calcium carbonate particle with poly­(allylamine hydrochloride) (PAH). The PAH’s positive charge served as a template for subsequent coating with ONB-modified DNA duplexes through electrostatic interactions and strand hybridization. After the assembly of the multilayered capsules, the calcium carbonate template was dissolved, resulting in hollow capsules housing dextran-conjugated doxorubicin (DOX-D). Upon uptake by MDA-MB-231 breast cancer cells, the capsule structure was dismantled upon UV-A light exposure, leading to the release of DOX-D for controlled cancer therapy. Notably, the efficacy of the drug was significantly enhanced by conjugating DOX to dextran, as the free DOX molecules were too small to escape from the capsules prior to photocleavage. This molecular photocleavage approach presents a novel method for the precise and remote release of various biomolecules for drug delivery with high temporal resolution. Additionally, Yang et al. applied a similar concept to develop aptamer-grafted hyperbranched polymers (HBP-DNA) by attaching dibenzocyclooctyne (DBCO)-modified aptamers to azide-functionalized HBPs featuring pendant ONB moieties (Figure 9B). These amphiphilic chains self-assembled to form HBP-DNA nanoparticles encapsulating DOX within hydrophobic cavities. Upon UV-A light exposure, the hydrophobic core of the drug delivery system experienced rapid reduction in hydrophobicity due to ONB moiety cleavage, leading to nanoparticle carrier disassembly and subsequent drug release. This light-triggered drug release demonstrated significant inhibition of cell proliferation in targeted cancer cells.^152^

**Figure 9:**
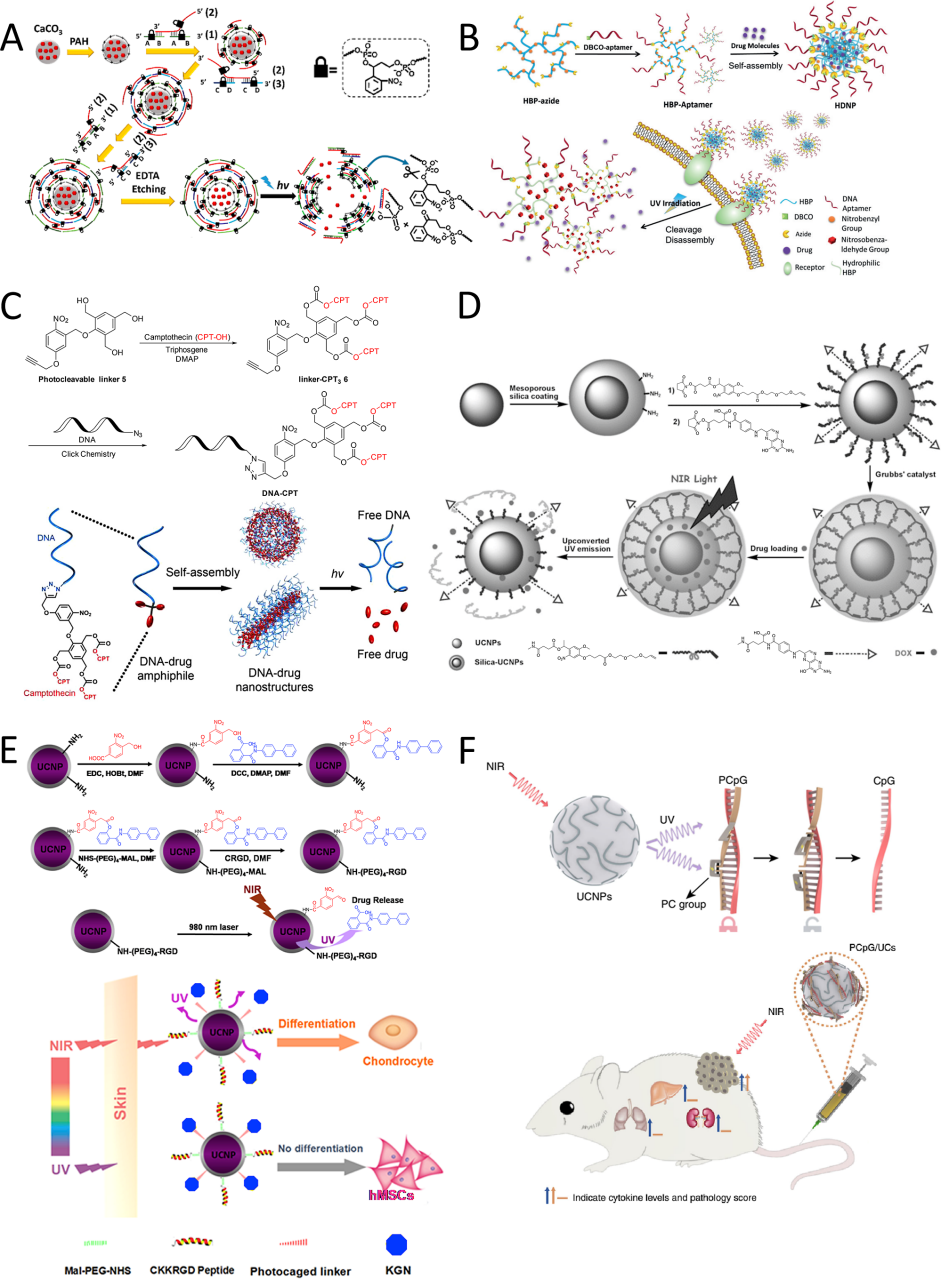
(A) Light-responsive DNA microcapsules designed for the precise release of DOX-D. Reproduced from ref 134 Copyright 2016 American Chemical Society. (B) Controlled and on-demand release of DOX from aptamer-grafted hyperbranched polymer within a cellular environment. Reproduced with permission from ref 152 Copyright 2018 John Wiley and Sons. (C) Formation schematic of DNA-CPT and its self-assembly to create DNA-drug nanostructures for UV-A light-triggered CPT release through irreversible micelle degradation. Reproduced from ref 142 Copyright 2015 American Chemical Society. (D) NIR light-managed DOX delivery utilizing OBN-caged mesoporous silica-coated UCNPs. Reproduced with permission from ref 167 Copyright 2013 John Wiley and Sons. (E) NIR-triggered KGN release from UCNPs for stem cell differentiation control and long-term in vivo tracking. Reproduced with permission from ref 168 Copyright 2016 Elsevier. (F) Integration of UCNPs with UV light-responsive PCpG to develop a photoactivatable immunodevice capable of releasing CpG oligonucleotides upon NIR irradiation, enabling refined temporal control over its immunoactivity. Reproduced from ref 169 Available under a CC-BY license. Copyright 2019 Chu et al. Strategies for light-regulated gene editing through caged CRISPR/Cas approaches primarily encompass the binding of a caged DNA protector to guide RNA, nucleobase-caged guide RNA, and terminus-modified photolabile crRNA.

Tan et al. developed and synthesized a DNA-camptothecin (CPT) amphiphile capable of self-assembling into micellar nanostructures with varied morphologies dependent on assembly conditions and the length of DNA strands.^142^ In this study, an ONB-based photocleavable linker **X**, functionalized with an alkyne group and a triphenol-based moiety, was employed to covalently conjugate with azide-functionalized DNA and three copies of hydrophobic CPT drug through a combination of classic click reactions and esterification reactions to create the building blocks. Upon activation by UV-A light, the DNAs were initially liberated from the nanostructure shell, exposing a decapped self-immolative drug core. This core subsequently underwent a rapid, spontaneous, and irreversible degradation process, leading to the release of free CPT molecules (Figure 9C). These DNA-CPT nanostructures exhibited improved stability against DNase I in comparison to free DNA. Moreover, the released model drug demonstrated comparable efficacy to unmodified free drugs against cancer cells, highlighting the potential of these structures as carrier-free delivery platforms.

In 1982, Seeman, a pioneer in DNA nanotechnology, introduced the concept that single-stranded DNA oligonucleotides could serve as a robust and versatile scaffold to construct nanoscale structures for in vitro and in vivo applications.^153–155^ Since then, researchers have designed and assembled addressable and biocompatible 2D and 3D DNA nanostructures capable of photoresponsive properties using various methods, aiming to create sophisticated smart drug delivery systems.^156–158^ To precisely regulate drug release efficiency, significant efforts have been dedicated to developing self-assembled DNA nanomaterials capable of converting photon energy into kinetic energy through bond cleavage within photolabile ONB derivatives. This innovative approach has led to a range of light-triggered responses, including changes in shape and size,^117^ structural switching,^81,159^ conformational alterations,^135,136^ aggregation and disaggregation.^145,146^ For instance, 3D DNA nanostructures and RNA pyramids have been utilized successfully as carriers for loading molecular cargoes, potentially enabling light-triggered release.^160–162^ Cargoes were attached to DNA building blocks via photocleavable ONB linkers. Following assembly, the molecular cargo was either confined within the DNA nanocage cavity or attached to the periphery of the RNA pyramid for potential delivery applications, ensuring their protection and deactivation. Upon UV-A light exposure, glutamate molecules were released from the DNA nanocage, subsequently activating neuron activity by monitoring glutamate-mediated calcium fluctuations in vulture neurons.^143^ In contrast, the phototriggered release of paclitaxel from the RNA pyramid led to heightened cytotoxicity in breast cancer cells compared to the RNA pyramid lacking photocleavable molecules.^163^ In another approach, Veetil et al. designed an icosahedral DNA nanocapsule capable of entrapping chemically modified dextran linked to cargoes via a DMNB linker for light-induced release of dehydroepiandrosterone (DHEA) to remotely induce neuronal activation.^164^ Upon photoirradiation at 400 nm, DMNB groups were cleaved, releasing the neurosteroid that diffused out of the nanocapsule. The sequestration of dextran within the nanocarrier prevented photocaged DHEA from interacting with cell surface receptors despite the carrier being anchored to the cell membrane. This study illustrated that integrating photoactivation technology with encapsulation offers a clean, artifact-free system for precise spatial and temporal control over neurosteroid signaling.

As discussed in Section 4.3, photon-upconversion materials have the unique capability to convert low-energy photons into higher-energy ones. Consequently, upconversion nanoparticles (UCNPs) offer a promising avenue for regulating drug release in nanodelivery systems.^165^ In a notable study spearheaded by Dcona et al., lanthanide-doped UCNPs were tailored with the anticancer drug doxorubicin (DOX) through covalent bonding via nitrobenzyl groups using click chemistry, a more robust approach than mere physisorption. This technique facilitated the controlled release of DOX upon NIR illumination.^166^ Another innovative strategy involves fine-tuning drug release at specific sites using a light-sensitive, cross-linked mesoporous silica-UCNP photocaging system.^167^ This system was engineered by linking 1-(2-nitrophenyl) ethyl photocaged oligo­(ethylene) glycol molecules, featuring a vinyl group at their ends, and attaching folic acids through condensation reactions. The subsequent intramolecular polymerization of the photocaged linkers, catalyzed by Grubbs’ second-generation catalyst in a ring-closing metathesis reaction, resulted in the formation of a cross-linked nanocarrier. This photocaged nanocarrier was then loaded with DOX for precise, NIR-triggered drug release in tumor cells (Figure 9D). These methodologies present promising avenues for precise cell imaging and regulated drug delivery within living organisms, offering advantages such as reduced photodamage and improved tissue penetration. Expanding on these benefits, Li et al. explored the application of this technique for targeted in vivo drug delivery. They devised a peptide-based UCNP@SiO_2_ nanocarrier by attaching kartogenin (KGN) to the nanoparticle core through a 3-nitrobenzoic acid linker (Figure 9E).^168^ KGN is known to enhance chondrogenesis in human mesenchymal stem cells (hMSCs) by interacting with intracellular signaling factors, thereby enhancing the uptake of UCNP nanocarriers by hMSCs. Upon NIR exposure, the release rate of KGN surged significantly from 0% to 60%. Notably, the controlled release of KGN from UCNPs proved more effective in guiding hMSC differentiation compared to direct KGN supplementation, enabling long-term tracking of hMSCs in vivo. Chu et al. also reported a NIR-responsive drug delivery system for CpG in vivo, showcasing the potential of this delivery platform to elicit a potent immune response at tumor sites while preserving immunity elsewhere in the body (Figure 9F).^169^

### 5.2 Gene Editing

The CRISPR/Cas system is a revolutionary tool in molecular biology that allows for precise editing of the genetic code in living organisms. The CRISPR system is a naturally occurring defense mechanism found in bacteria and archaea that helps protect them from viral attacks. Cas enzymes associated with the CRISPR system are responsible for cutting DNA at specific locations. When the CRISPR/Cas system is introduced into a cell, the gRNA directs the Cas enzyme to the target gene where it makes a precise cut in the DNA. This cut can then be repaired by the cell’s natural DNA repair mechanisms, potentially leading to gene knockout, gene insertion, or gene modification. Its ease of use, efficiency, and precision have made it a widely adopted tool in molecular biology and biotechnology. Altered guide RNA has the potential to enhance the specificity, stability, and editing effectiveness of the CRISPR/Cas system.

Various strategies can be employed to regulate the activity of the CRISPR/Cas system by manipulating the guide RNA (gRNA). One approach involves the introduction of a caged DNA protector (DNA-p) that forms full or partial complementary pairs with the short guide RNA (sgRNA). Jain et al. devised a UV-responsive CRISPR/Cas9 system utilizing a DNA-p that was altered with three ONB-linkers within a six-nucleotide interval. When exposed to UV-A light, the DNA-p breaks apart due to the ONB-linker, reducing its stability in binding to the sgRNA targeting GFP, CD71, or CD33, thereby activating the CRISPR/Cas9 system. This concept led to the creation of the CRISPRplus technique, demonstrating the ability to simultaneously target multiple sequences (Figure 10A).^137^ Moreover, a photocontrolled single-step assay was developed to resolve the conflict between CRISPR detection and nucleic acid isothermal amplification systems.^138,139^ Although showcasing the effectiveness of the gRNA activity regulation technique, this method comes with specific constraints. Precise fine-tuning of the gRNA to DNA-p ratio is crucial to fully suppress sgRNA activity. Furthermore, this method impacts the binding efficacy between sgRNA and Cas protein, resulting in reduced stability of Cas proteins and sgRNAs within the reaction setup. Recently. Abe et al. showcased the utilization of a ring-shaped DNA origami structure to conceal the activity of Cas9.^140^ In their design, a singular sgRNA was engineered to pinpoint a specific sequence on the substrate dsDNA. Additionally, an additional RNA linker was devised to attach to the 3′-end of the sgRNA, hybridizing with a longer complementary DNA strand modified with ONB to affix to the nanoring via 24 anchoring strands. The photocleavable linker was efficiently excited by photoirradiation at 350 nm within 5 min, leading to the photoinduced liberation of the Cas9/sgRNA complex for the cleavage of the target DNA. This setup allows for the systematic integration of enzymes into the ring structure for purification, followed by their controlled activation via light-induced release.

**Figure 10:**
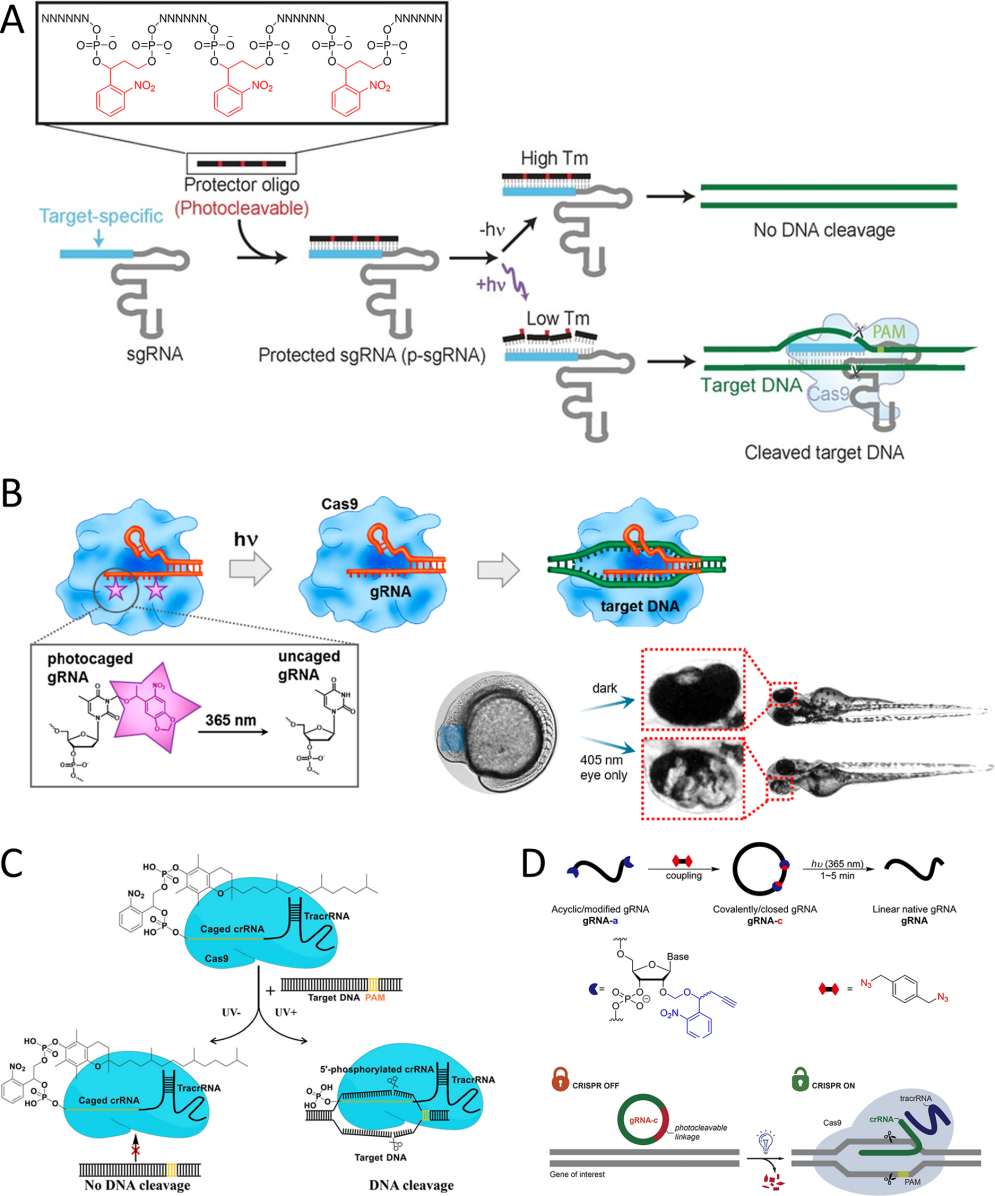
(A) CRISPR-plus enables photoactivatable blockade of Cas9-mediated DNA targeting by incorporating an ONB-caged DNA protector. Reproduced with permission from ref 137 Copyright 2016 John Wiley and Sons. (B) Spatiotemporally controlled CRISPR/Cas-based genomic regulation in zebrafish through the photouncaging of NPOM-based gRNAs. Reproduced from ref 147 Copyright 2020 American Chemical Society. (C) Photoregulation of CRISPR/Cas9-induced gene editing using terminus-modified ONB-caged crRNA for endogenous VEGFA and exogenous EGFP in HEK293T cells. Reproduced with permission from ref 144 Copyright 2020 John Wiley and Sons. Figure (D) Development and synthesis of circular single-stranded gRNAs for spatiotemporal control of CRISPR/Cas9 editing. Reproduced with permission from ref 150 Copyright 2022 John Wiley and Sons.

For enhanced suppression of gRNAs, a method involving photochemically activated gRNAs integrated into caged compounds has been employed to regulate the functionality of the CRISPR/Cas system. Caged molecules such as NPOM^170^ and DMNEC,^22^ modified either on the nucleobases or at the 2′-OH position of ribose^171^ on sgRNA, introduce steric hindrance effects that can effectively silence the CRISPR/Cas system by obstructing the interaction of the crRNA with its target. Upon UV-A exposure, the caged crRNA is uncaged, leading to a swift restoration of its functionality. Moroz-Omori et al. reported a series of innovative nucleobase-caged sgRNAs targeting ZsGreen, HPRT, IL1R2, Tomato, and Albino for spatiotemporal photoregulation of CRISPR/Cas9 genome editing and the activation of target gene transcription in HEK293FT cells and zebrafish embryos (Figure 10B). They highlighted the advantages of simple synthesis and purification of caged oligonucleotides compared to engineered caged Cas9 proteins.^147^ The caged crRNA/tracrRNA duplex was designed with the incorporation of two photocleavable NPOM-modified dT groups at the specific target region of the guide crRNA to impede the recognition of the genome target DNA sequence before irradiation. Following photolysis with 365 nm light, the photoswitchable crRNA was completely uncaged, reinstating the activity of CRISPR/Cas9-mediated genomic DNA cleavage. Deiters et al. have implemented a similar concept to develop another nucleobase-caged guide RNA that integrates four NPOM-modified rU or rG phosphoramidites evenly within the base-pairing region of the guide RNA. This guide RNA targets DsRed, EGFP, CTNNb2, or SLC24 A5 for the optical regulation of CRISPR/Cas9 activity with spatial and temporal precision in mammalian cells and zebrafish.^148^ Additionally, a novel photocontrolled single-step nucleic acid detection system, based on CRISPR/Cas12a, has recently been successfully pioneered by Hu et al.^149^ In comparison to previous approaches, the current photocontrolled strategy streamlines the development of more robust one-pot CRISPR assays. It is more straightforward, as it utilizes the same reagent preparation process as conventional CRISPR assays, faster due to the elimination of the RNA hybridization step, more stable, and cost-effective. While this current method shows promise, its efficacy is impeded by limited generalizability and the need for laborious trial-and-error screening of the position and number of NPOM modifications in response to variations in the target sequence.

Deiters et al. have pioneered the development of a caged CRISPR-Cas9 system, utilizing protein engineering techniques in addition to caged gRNA. This system enables the photoregulation of genome editing and gene expression, targeting both exogenous GFP/RFP and endogenous CD71 genes within live HEK293T cells.^172^ Caged Cas9 protein underwent site-specific modification with an NPOM caging group at the crucial K866 lysine residue, integral for Cas9-mediated gene editing. The photocaged group attached to lysine could be selectively cleaved, restoring CRISPR-Cas9 functionality upon exposure to UV-A light for 2 min.

Zhang et al. have introduced a novel caged CRISPR/Cas9 genome editing system targeting EGFP or VEGFA. This system involves terminus-modified photolabile crRNA with a single vitamin E or cholesterol adjustment at the 5′ terminus of the crRNA. This modification functions by obstructing the interaction between the ribonucleoprotein and the target DNA. Upon irradiation with 365 nm light, the activation of CRISPR/Cas9-mediated genomic DNA editing is effectively triggered (Figure 10C). The caged crRNA, modified with vitamin E or cholesterol, demonstrates significant efficacy in the photoregulation of CRISPR/Cas9-induced gene editing for both endogenous VEGFA and exogenous EGFP in HEK293T cells.^144^ In addition to photocaging sgRNAs, another effective strategy for achieving precise spatiotemporal control of CRISPR/Cas-mediated gene editing involves restricting the conformation of sgRNAs. Zhang et al. developed an RNA-clamp approach that silences gRNA activity by enzymatically cross-linking two specific guanine residues within the gRNA using a photocleavable nitrobenzyl-based cross-linker.^141^ This intramolecular clamp alters the normal structure of the gRNA, preventing its recognition and base pairing with the target sequence. Upon exposure to light in the 356–390 nm range, the cross-linker is cleaved with high spatial and temporal precision, thereby restoring CRISPR/Cas9 gene editing activity. In addition to the ONB group, the researchers also employed a [7-(diethylamino)­coumarin-4-yl]-methyl (DEACM) cross-linker. This alternative design, responsive to different wavelengths of light, enables multiplexed photoactivated gene editing in mammalian cells. Additionally, Sun et al. further advanced the control of CRISPR/Cas activity by designing circular guide RNAs (gRNA-c) through the incorporation of two or three photolabile groups followed by intramolecular cyclization (Figure 10D).^150^ Specifically, the (1′-propargyl-*o*-nitrobenzyloxy)­methoxyl (NPBOM) group, containing terminal alkyne functionalities, was introduced at two strategically chosen adenosine residues—one in the spacer region and the other in the duplex region of the gRNA. These modified sites were then covalently linked via a copper-catalyzed azide–alkyne cycloaddition (CuAAC) reaction with a bifunctional azide linker, 1,4-bis­(azidomethyl)­benzene, to generate a circularized gRNA structure. In this conformation, the gRNA is unable to recognize its target and remains inactive until UV-A light exposure (1–5 min), which cleaves the photolabile groups and restores the linear, functional form of the gRNA. This method offers improved resistance to degradation and more effective suppression of gRNA activity compared to conventional linear gRNAs, which often require extensive screening and optimization. However, achieving complete reaction efficiency in both cyclization and clamping strategies remains a technical challenge. Additionally, these structural modifications can lead to unintended intermolecular interactions. To minimize the influence of incomplete or unmodified gRNAs on experimental results, purification of the reaction products is necessary—adding complexity to the overall experimental workflow.

In addition to strategies that activate the CRISPR/Cas system, developing methods to deactivate it with high spatiotemporal precision is equally important. Persistent CRISPR/Cas activity within cells can lead to undesirable effects such as off-target editing, genotoxicity, chromosomal translocations, and even malignancy. To address this, Carlson-Stevermer et al. introduced the CRISPRoff approach, which enables light-induced degradation of gRNA to switch off CRISPR activity (Figure 11A). In this method, two photocleavable residues containing ONB groups were incorporated at distinct positions within the gRNA backbone. Upon UV-A irradiation, these groups are cleaved, leading to fragmentation of the gRNA and loss of its function.^96^ This technique has proven effective across multiple genomic targets and cell lines, offering a powerful means to precisely control both the activation and deactivation of CRISPR-mediated gene editing. Furthermore, Zou et al. engineered a photocleavable gRNA by incorporating an ONB linker at or before the 15th nucleotide position of a full-length crRNA. Upon light irradiation, this modification rapidly converts the gRNA from a cleavage-competent full-length form to a truncated, cleavage-deficient form which is effectively deactivating both Cas9 nuclease and base editor activity within seconds (Figure 11B).^79^

**Figure 11:**
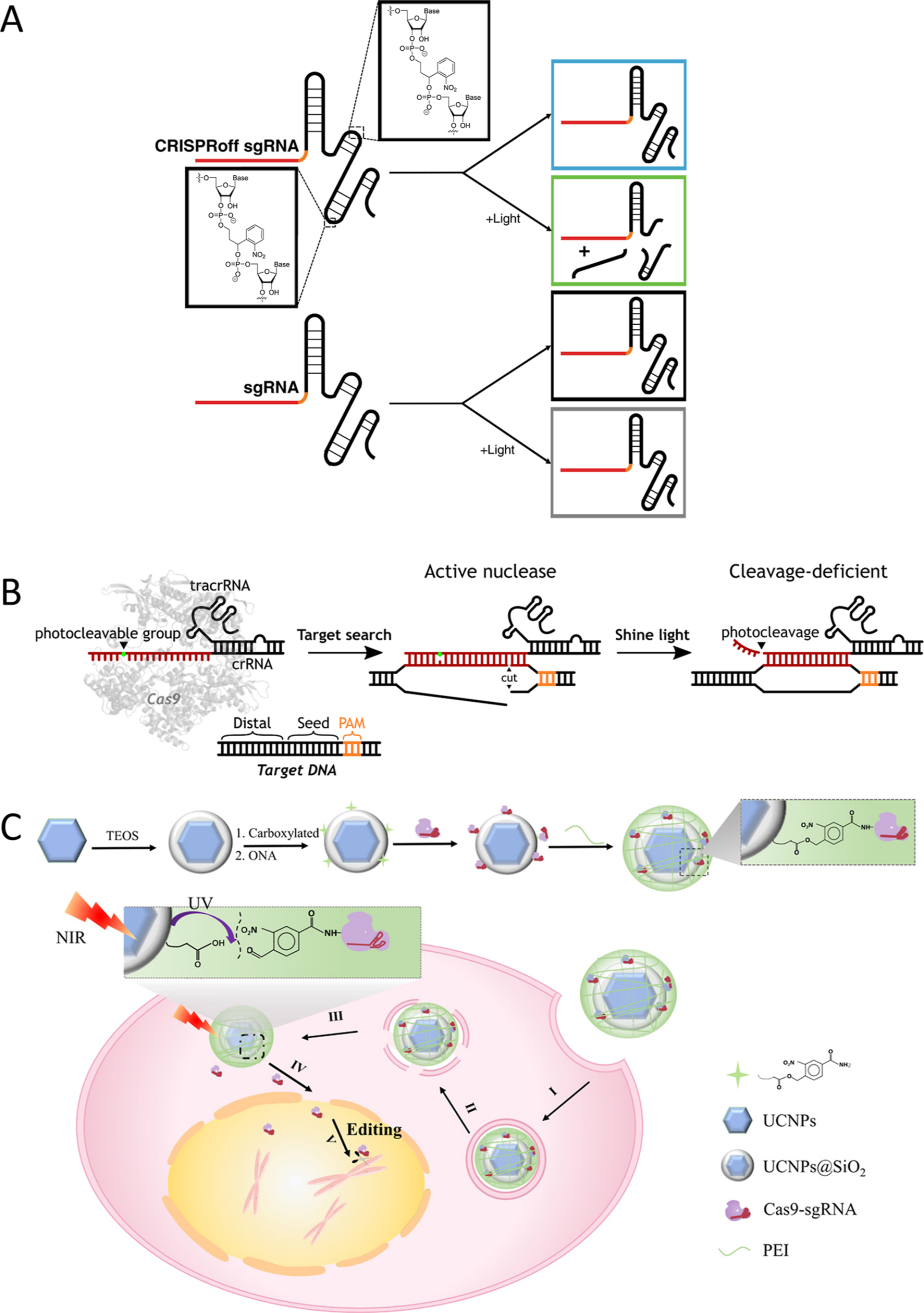
(A) Representation of CRIPSRoff dual-breakage (DB) sgRNAs involving ONB linkers at nucleotide positions 57 and 74. Upon light exposure, the DBsgRNAs fragment and lose functionality. Reproduced from ref 96 Available under a CC-BY license. Copyright 2020 Carlson-Stevermer et al. (B) Design of a NOB linker-caged gRNA at or before the 15th nucleotide position for rapid conversion from a cleavage-competent full-length form to a truncated, cleavage-deficient form, deactivating Cas9 nuclease and base editor activity swiftly. Reproduced with permission from ref 79 Copyright 2021 Elsevier. (C) Design and working principles of the UCNP-based CRISPR-Cas9 delivery system for gene editing: cellular uptake (I and II), endosomes escape (III), Cas9-sgRNA release after NIR irradiation (IV), and translocation into the nucleus to bind with target DNA, initiating the DNA double-strand break (V). Reproduced from ref 174 Available under a CC-BY NC license. Copyright 2019 Pan et al.

In current photocontrolled crRNA technologies, a key challenge arises from the necessity of high-energy UV-A radiation within the 365–400 nm wavelength range to disrupt the ONB-linker over extended periods. This requirement not only poses risks of skin and ocular damage but also raises concerns regarding potential genetic mutations, thereby jeopardizing the integrity of the experimental subject.^173^ Moreover, UV operation mandates specialized facilities and stringent safety measures, complicating experimental procedures. Consequently, the adoption of longer-wavelength activation with reduced energy input has proven instrumental in enhancing the efficiency of gene editing applications. A recent breakthrough in cancer therapy has focused on the development of a NIR-activated caged CRISPR-Cas9 system utilizing NIR-to-UV UCNP. In this novel system, the CRISPR-Cas9 protein underwent covalent modification with a dense silica shell on the UCNP surface, employing a photolabile group (Figure 11C).^174^ Subsequently, the UCNP-Cas9 complex was coated with PEI to facilitate escape from endosomes. Upon exposure to 980 nm NIR light, the Cas9 protein/guide RNA duplexes targeting PLK-1 were released from the particles, translocated into the nucleus to locate the target DNA locus, and initiated DNA double-stranded breaks for photomodulation of genome editing, tumor growth, and progression using upconverted UV-A light irradiation, both in vivo and in vitro. Essentially, upconversion nanomaterials are pivotal in advancing UV/visible light-cleavable photocages by enabling the utilization of safer NIR light to trigger the release of bioactive molecules through NIR-to-UV conversion. This capability significantly enhances the potential of such systems for a broad spectrum of biological and medical applications.^175–177^

## 6 Summary and Outlook

Light-responsive nucleic acids represent a highly promising class of biomaterials with broad applications in biomedicine. Their structural configuration, hybridization, and dehybridization behaviors can be precisely regulated by tuning light parameters such as wavelength, intensity, and exposure time. Through various chemical strategies, photocleavable ONB-based derivatives can be incorporated into specific sites of nucleic acid sequences, enabling controlled modulation of their functions. The biostability of ONB-based photolabile protecting groups (PPGs) is crucial for their effectiveness in biomedical applications involving nucleic acid nanostructures. These PPGs must exhibit robust resistance to enzymes, pH variations, and temperature fluctuations to ensure their functionality within cellular environments. ONB-based PPGs play a pivotal role in providing temporal control over the stability of nucleic acid nanostructures. By exposing these nanostructures to specific wavelengths of light, the cleavage of ONB groups can be triggered, inducing structural modifications in the nanostructures. This precise temporal control enables tailored adjustments to the stability of the nanostructures in a controlled manner. Moreover, the incorporation of ONB groups can bolster the overall stability of nucleic acid nanostructures. By selectively shielding key functional groups within the nucleic acids, ONB groups act as barriers against undesired interactions or degradation processes. This protective mechanism is instrumental in preserving the structural integrity of the nanostructures across diverse environmental conditions. Furthermore, the ability to selectively eliminate ONB groups through light exposure facilitates the controlled release of functional groups or payloads attached to the nucleic acid nanostructures. This controlled release capability is particularly vital for targeted drug delivery applications, where the precise release of payloads at specific locations or time points is essential. The versatility of photocleavable nucleic acids/nanostructures, enabled by ONB-based PPGs, holds immense value in applications such as controlled drug delivery and gene editing. This adaptability and controlled release mechanism make them highly advantageous for a range of biomedical applications, where precision and tailored functionality are paramount.

Despite significant progress and successful proof-of-concept demonstrations, most studies remain confined to in vitro settings. A major limitation is the prevalent dependence on UV-A light for activation, particularly in CRISPR/Cas systems, which hinders in vivo applicability due to UV’s limited tissuepenetration and potential cytotoxicity. Moreover, current light-regulated CRISPR systems do not yet achieve complete activation or deactivation efficiency. While increasing light dosage may enhance response, it often comes at the cost of cellular damage. Therefore, there is a growing interest in using longer-wavelength light sources, which offer better tissue penetration and lower toxicity. For in vivo applications, it is crucial to develop biocompatible, photocleavable ONB-based oligonucleotides with high aqueous solubility, efficient cellular uptake, excellent photostability, and minimal cytotoxicity. Achieving these properties remains a significant challenge. Efficient intracellular delivery of these photoresponsive oligonucleotides is another critical hurdle. Notably, Dai et al. have shown that incorporating photosensitive oligonucleotides into self-assembling DNA nanostructures can facilitate effective cellular delivery with minimal toxicity—an important step toward practical biomedical applications.^117^ Recently, we reported an ANBP-based DNA system designed for potential gene therapy, featuring a photocleavable DNA nanocarrier activated by 415 nm light.^151^ Although this wavelength does not penetrate as deeply as NIR light, it remains suitable for localized therapies, such as retinal treatments. For example, studies by Wang et al. and Brandhorst et al. have demonstrated successful phototriggered drug release^178^ and gene expression^179^ in mice using 400 nm light. Although our ANBP derivative has shifted absorption out of the UV-A range and exhibits two-photon uncaging capabilities at 740–800 nm—within the biological transparency window, the uncaging efficiency of ONB-conjugated oligonucleotides is often lower than that observed in small molecules like glutamate, due to factors such as steric hindrance, sequence-dependent chemical environments, and structural constraints that affect reaction kinetics. To enhance the photon uncaging efficiency of ONB-conjugated oligonucleotides, future efforts should focus on optimizing conjugate design, exploring alternative caging groups, and adjusting experimental parameters to mitigate the factors limiting uncaging performance.

Additionally, DNA’s vulnerability to enzymatic degradation by nucleases necessitates stringent handling and storage conditions, contributing to elevated costs in fabrication and maintenance of DNA-based soft materials. To address these challenges, alternative nucleic acid chemistries—such as xeno nucleic acids (XNAs)—are being explored as substitutes for conventional caged oligonucleotides.^180^ Among these, synthetic threose nucleic acid (TNA) has emerged as a promising option, in which it is an RNA-like polymer composed of a four-carbon threose sugar backbone linked by phosphodiester bonds at the 2′- and 3′-positions of the sugar ring.^181^ Compared to natural DNA, we have successfully indicated that TNAs demonstrate remarkable resistance to enzymatic degradation in serum and cellular extracts, as well as exceptional stability under extreme pH conditions.^182^ They could be safely administered into animal models via tail vein injection, without inducing any pathological changes or severe functional and structural damage to the renal systems.^183^ These attributes make TNA a highly viable alternative to traditional antisense oligonucleotides for in vivo gene silencing and tumor suppression, owing to its enhanced specificity and stronger binding affinity toward complementary RNA strands.^184,185^ More recently, we have leveraged TNA as a structural component in the design of miRNA biosensors for real-time, intracellular nucleic acid detection and imaging.^186,187^ Given these versatile and robust properties, TNA presents a compelling platform for the development of light-responsive systems.^188^ We envision integrating PPGs into TNA-based architectures to explore their potential in light-activated biomedical applications. Although challenges remain in translating light-responsive nucleic acid nanosystems into clinical practice, ongoing advancements in regulatory mechanisms and performance optimization are expected to significantly enhance their utility in biomedical research and therapeutic interventions.
